# Towards accurate and unbiased imaging-based differentiation of Parkinson’s disease, progressive supranuclear palsy and corticobasal syndrome

**DOI:** 10.1093/braincomms/fcaa051

**Published:** 2020-04-27

**Authors:** Marta M Correia, Timothy Rittman, Christopher L Barnes, Ian T Coyle-Gilchrist, Boyd Ghosh, Laura E Hughes, James B Rowe

**Affiliations:** f1 MRC Cognition and Brain Sciences Unit, University of Cambridge, UK; f2 Department of Clinical Neurosciences and Cambridge University Hospitals NHS Foundation Trust, University of Cambridge, Cambridge, UK; f3 Janelia Research Campus, Howard Hughes Medical Institute, USA; f4 Wessex Neurological Centre, University Hospital Southampton NHS Foundation Trust, UK; f5 Danish Research Centre for Magnetic Resonance, Centre for Functional and Diagnostic Imaging and Research, Copenhagen University Hospital Hvidovre, Denmark

**Keywords:** Parkinson’s disease, progressive supranuclear palsy, corticobasal degeneration syndrome, magnetic resonance imaging, support vector machine

## Abstract

The early and accurate differential diagnosis of parkinsonian disorders is still a significant challenge for clinicians. In recent years, a number of studies have used magnetic resonance imaging data combined with machine learning and statistical classifiers to successfully differentiate between different forms of Parkinsonism. However, several questions and methodological issues remain, to minimize bias and artefact-driven classification. In this study, we compared different approaches for feature selection, as well as different magnetic resonance imaging modalities, with well-matched patient groups and tightly controlling for data quality issues related to patient motion. Our sample was drawn from a cohort of 69 healthy controls, and patients with idiopathic Parkinson’s disease (*n *= 35), progressive supranuclear palsy Richardson’s syndrome (*n *= 52) and corticobasal syndrome (*n *= 36). Participants underwent standardized T1-weighted and diffusion-weighted magnetic resonance imaging. Strict data quality control and group matching reduced the control and patient numbers to 43, 32, 33 and 26, respectively. We compared two different methods for feature selection and dimensionality reduction: whole-brain principal components analysis, and an anatomical region-of-interest based approach. In both cases, support vector machines were used to construct a statistical model for pairwise classification of healthy controls and patients. The accuracy of each model was estimated using a leave-two-out cross-validation approach, as well as an independent validation using a different set of subjects. Our cross-validation results suggest that using principal components analysis for feature extraction provides higher classification accuracies when compared to a region-of-interest based approach. However, the differences between the two feature extraction methods were significantly reduced when an independent sample was used for validation, suggesting that the principal components analysis approach may be more vulnerable to overfitting with cross-validation. Both T1-weighted and diffusion magnetic resonance imaging data could be used to successfully differentiate between subject groups, with neither modality outperforming the other across all pairwise comparisons in the cross-validation analysis. However, features obtained from diffusion magnetic resonance imaging data resulted in significantly higher classification accuracies when an independent validation cohort was used. Overall, our results support the use of statistical classification approaches for differential diagnosis of parkinsonian disorders. However, classification accuracy can be affected by group size, age, sex and movement artefacts. With appropriate controls and out-of-sample cross validation, diagnostic biomarker evaluation including magnetic resonance imaging based classifiers may be an important adjunct to clinical evaluation.

## Teaser

We address the methodological issues affecting recent studies that combine imaging data and machine learning for the differential diagnosis of parkinsonian disorders. Our results confirm that potential for accurate classification using optimal feature selection, but matching groups and data quality are essential to avoid biased classification.

## Graphical Abstract

**Graphical Abstract:**
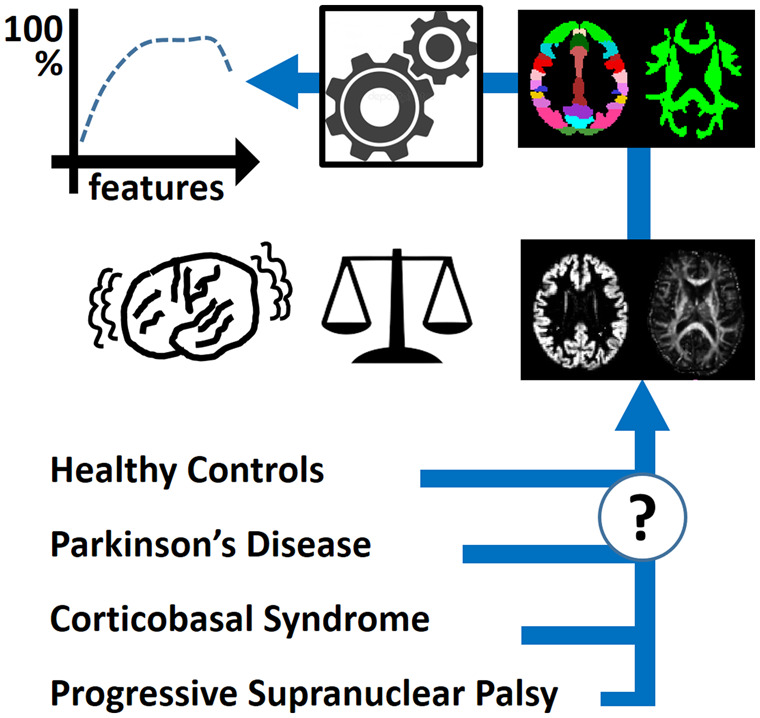

## Introduction

The early and accurate differentiation of parkinsonian disorders poses a challenge for clinicians and trialists, which will become critical with the advent of disease modifying therapies (van Eimeren *et al.*, 2019). Early symptoms and signs often overlap between idiopathic Parkinson’s disease, progressive supranuclear palsy (PSP) and corticobasal syndrome (CBS, and its pathological counterpart corticobasal degeneration, CBD). Parkinson’s disease is the most common form of parkinsonism, with approximately 140 cases per 100 000 (Porter *et al.*, 2006) whereas PSP and CBS are each approximately 3 per 100 000 (Coyle-Gilchrist *et al.*, 2016). Misdiagnosis of PSP and CBS is common, often as Parkinson’s disease, taking on average nearly 3 years from initial symptoms to diagnosis, while many cases remain undiagnosed.

There is a pressing need for reliable biomarkers to differentiate these disorders, not only to aid diagnosis in early cases, but to monitor progression in trials and to support *ante mortem* studies of pathogenesis (van Eimeren *et al.*, 2019). Biomarkers should be objective and observer-independent, reproducible, informative about the underlying biology and ideally non-invasive. Candidate biomarkers for parkinsonian disorders have included cognitive tests (Pillon *et al.*, 1995; Aarsland, 2003; Rittman *et al.*, 2013) and assays of cerebrospinal fluid, serum or urine such as neurofilament light chain (Jabbari *et al.*, 2017; Constantinescu *et al.*, 2019; Jabbari *et al.*, 2020), supplementing those clinical features that have high clinicopathological correlations (Alexander *et al.*, 2014; Respondek *et al.*, 2017; Gazzina *et al.*, 2019).

Magnetic resonance imaging (MRI) provides a set of potential biomarkers (Whitwell *et al.*, 2017), with the advantages of being non-invasive, widely available and versatile. Multiple MRI methods have the potential to inform about the underlying neural systems and the changes resulting from specific pathologies. Pathognomic radiological signs have been reported, such as the ‘mickey mouse’ and ‘hummingbird’ signs of mid-brain atrophy in PSP, but although they have good specificity, sensitivity is limited, especially in early stage disease when there would be most to gain from disease modifying therapies.

Automated methods have been developed using volumetric or intensity change in grey matter (GM), for example voxel-based morphometry (VBM). Most VBM studies of grey matter in degenerative parkinsonian syndromes have compared patients to healthy controls (Brenneis *et al.*, 2004; Cordato *et al.*, 2005; Summerfield *et al.*, 2005; Beyer *et al.*, 2006; Ghosh *et al.*, 2012; Yarnall *et al.*, 2014). A few have compared patient groups against each other (Price *et al.*, 2004; Boxer *et al.*, 2006), or compared subgroups within each disorder, according to cognitive impairment (Paviour *et al.*, 2006; Mak *et al.*, 2015) or neuropsychiatric symptoms (Ghosh *et al.*, 2012; Yao *et al.*, 2014). White matter (WM) changes have also been described, using VBM or diffusion tensor imaging (DTI) measures such as the fractional anisotropy (FA) and mean diffusivity (MD). Differences are observed for Parkinson’s disease versus controls (Yoshikawa *et al.*, 2004; Zhang *et al.*, 2011; Rae *et al.*, 2012; Goveas *et al.*, 2015), Parkinson’s disease versus PSP (Seppi *et al.*, 2003) and Parkinson’s disease versus CBS (Boelmans *et al.*, 2010). A meta-analysis of 43 DTI studies in parkinsonian syndromes (Cochrane and Ebmeier, 2013) suggested the potential of diffusion-weighted imaging to improve the differential diagnosis of parkinsonism. However, accuracy was often not greater than clinical criteria, sample sizes were often small, and the utility for single subject decision-making was limited.

We propose that better classification can be achieved by using statistical classifiers such as support vector machines (SVM). Mulitvariate data features from a training set of data (subjects) can be used to build a model to classify a new dataset (one or more new subjects). In addition to individual subject classification, these methods can identify which features underlie the classification (i.e. indicative of relevant pathological features) and indices of confidence or typicality that could be used to assess progression. Statistical classifiers have been applied to several neurological and psychiatric disorders, including schizophrenia (Caan *et al.*, 2006; Ingalhalikar *et al.*, 2010), Alzheimer’s disease, frontotemporal dementia (Davatzikos *et al.*, 2008) and autism spectrum disorder (Ingalhalikar *et al.*, 2010, 2011; Bloy *et al.*, 2011). Haller *et al.* (2012) used DTI data from 17 Parkinson’s disease patients and 23 patients with ‘atypical parkinsonism’ (including typical PSP and multiple system atrophy). Using tract based spatial statistics (TBSS), a non-linear SVM algorithm, and a 10-fold cross-validation, classification between Parkinson’s disease and other patients was accurate (97.5 ± 7.5%, depending on the number of features used for model training). In combination with manual regions-of-interest selection, classification accuracies >95% were also achieved by Prodoehl *et al.* (2013) in binary differentiation of Parkinson’s disease and PSP. T1-weighted MRI can also support binary classification >85% (Focke *et al.*, 2011; Salvatore *et al.*, 2014).

Unfortunately, whilst previous studies have demonstrated successful differential diagnosis of parkinsonism, significant limitations and methodological questions remain. First, many studies have used poorly matched groups in terms of age or clinical variables, and different numbers of subjects in each group. The latter is of particular concern because commonly used statistical classifiers which minimize the classification error (including SVMs), are liable to inflate accuracy from unbalanced datasets (see for example, He and Garcia, 2009; Tang *et al.*, 2009).

A second problem relates to the selection of features for the classifier. For example, previous studies have used either mean values from specified regions or individual voxel data, including manual selection with operator dependence. In addition, studies have rarely compared different MRI modalities to assess whether T1-weighted or diffusion-weighted imaging (DWI) are most useful. While most studies using T1-weighted data have used GM volume as feature type, cortical thickness is a valuable alternative (for example Gao *et al.*, 2018; Wilson *et al.*, 2019).

A third problem concerns the validation of results, which is challenging with small group sizes. Most studies have included small numbers of subjects, and therefore employed cross-validation techniques. However, the use of the same subjects for training and validation is controversial and may inflate classification accuracies. A more conservative approach is to split the data in two independently acquired groups: one for training and the other for validation (Salvatore *et al.*, 2014), or to use independent datasets for validation.

Finally, most studies have failed to consider how different levels of motion during the MRI acquisition affect classification accuracies. This issue is particularly important when working with patients with movement disorders. Head motion results in artefacts and smoothing of MRI data. Different levels of motion across groups could significantly contribute to classifier’s apparent success in separating patient groups.

In the present study, we aimed to address these four methodological issues in the context of differential diagnosis of Parkinson’s disease, CBS and the Richardson’s syndrome variant of PSP (PSP-RS). Specifically, we compare three equal-sized and closely matched groups of patients; we used automatic feature selection of grey and white matter signals; and we undertook an initial leave-two-out cross-validation followed by validation in an independent data set. The comparison of well-matched groups, with automatic feature selection is a challenge for imaging markers, but one that is necessary to develop unbiased and useful clinical research tools.

## Methods

### Subjects

Our analysis sample was drawn from a cohort of 69 healthy controls (mean age 67.3 years, range 51–84), 35 people with idiopathic Parkinson’s disease (mean age 66.9 years, range 46–76, UK Parkinson’s disease brain bank criteria), 52 people with probable PSP-RS [mean age 71.9 years, range 51–92, MDS clinical diagnostic criteria for PSP-Richardson’s syndrome (Höglinger *et al.*, 2017)] and 36 people with probable CBS [mean age 66.9 years, range 39–88 (Armstrong *et al.*, 2013)], with UPDRS-III motor subscale for all patients.

For the cross-validation analysis (see below), 19 cases per group were selected so as to match for age, sex, and MRI motion, with similar UPDRS-III scores in the patient groups. Local Ethical Committee approval and written informed consent were obtained. All participants had mental capacity to consent under UK law.

### MRI data acquisition

Diffusion and T1-weighted MRI data were acquired for all subjects using a 3 T Siemens Tim TRIO scanner at the Wolfson Brain Imaging Centre. Diffusion MRI data were acquired with a twice refocused spin echo (TRSE) sequence (Reese *et al.*, 2003). Diffusion sensitising gradients were applied along 63 non-collinear directions with a b-value of 1000 s/mm^2^, together with one acquisition without diffusion weighting (b = 0). The remaining imaging parameters were: TR = 7800 ms, TE = 90ms, matrix = 96 × 96, field of view (FoV)=192 × 192 mm, slice thickness = 2 mm without gap, interleaved slice acquisition, and the PAT mode was GRAPPA with an acceleration factor of 2. A high resolution 3D T1-weighted MPRAGE image was also acquired (TR = 2300 ms, TE = 2.98 ms, FoV = 256 × 240 mm, matrix = 256 × 256, slice thickness = 1 mm).

### Quality assurance and exclusion criteria

MRI data in general, and diffusion MRI in particular, can suffer from significant distortions in the presence of head motion. Given the motor deficits associated with parkinsonism, metrics of motion are especially important to ensure the quality of the data across control and patient groups. Estimating the amount of motion in 3D MPRAGE images is not trivial. We used SPM12 (www.fil.ion.ucl.ac.uk/spm/) to estimate the level of smoothness associated with the MPRAGE images of each subject. Because of its induction of spatially correlated noise, motion is expected to correlate with the inherent smoothness in the data. Firstly, we performed full image segmentation using the *Segment* tool in SPM12 (Ashburner and Friston, 2005). Secondly, the *spm_estimate_smoothness* function was used to estimate the inherent smoothness associated with soft tissue outside the brain, cerebral spinal fluid (CSF) and bone. This function returns a spatial smoothness estimator based on the variances of the normalized spatial derivatives as described in (Kiebel *et al.*, 1999). The estimated smoothness values were then compared across controls and patients, and significant outliers (>2 standard deviations from the mean) were visually inspected and removed from further analysis (data quality was evidently poor for all subjects flagged by this metric).

For the diffusion MRI data, we estimated motion artefacts in two ways. Firstly, we used the *eddy_correct* function in FSL v5.0.9 (www.fmrib.ox.ac.uk/fsl) to perform affine registration between each diffusion weighted volume and the b = 0 image. The output log files from *eddy_correct* were used to estimate the absolute displacement between each diffusion MRI volume and the b = 0 images, as well as the relative displacement between a given volume and its predecessor. Significant outliers (>2 standard deviations from the mean) on either metric were identified and removed from further analysis. Subjects were also excluded if they moved more than 3 mm (1.5 × voxel size) between any two diffusion MRI volumes. Secondly, we used an automated method for detection of striping patterns in the data (Neto-Henriques *et al.*, 2016). Striping artefacts are caused by spin history and are a common consequence of head motion when interleaved MRI acquisitions are used. Subjects with more than five volumes affected by striping artefacts were excluded.

### Cross-validation and validation groups

The remaining subjects were divided into two subgroups: a cross-validation group and an independent validation group. The subjects included in the cross-validation group were selected to satisfy the following criteria:

- Equal numbers of subjects across the four control/patient groups
- No significant differences in motion metrics across the four control/patient groups
- No significant age or sex differences across the four control/patient groups
- UPDRS-III scores matched for all three patient groups

All remaining subjects who had not been excluded by the motion quality control metrics made up the validation group.

### Pre-processing of MRI data

The T1-weighted MPRAGE images were segmented and normalized into MNI space using SPM12. Firstly, the MPRAGE images were segmented into grey and white matter maps using *Segment* (Ashburner and Friston, 2005). For this step, six tissues types were considered (grey matter, white matter, CSF, bone, soft tissue outside the brain, and air and other signals outside the head). Total intracranial volume (TIV) was estimated by summing probability maps for GM, WM and CSF in native space. Segmentation was then followed by DARTEL (Diffeomorphic Anatomical Registration Through Exponentiated Lie Algebra) (Ashburner, 2007), an algorithm which increases the accuracy of inter-subjects alignment by modelling the shape of each brain using three parameters per voxel, and generating an increasingly sharp average template over several iterations. Finally, the sixth iteration of the DARTEL template was used to generate spatially normalized and Jacobian scaled grey matter images in MNI space (Mechelli *et al.*, 2005; Ashburner, 2009).

In parallel, T1-weighted data were pre-processed using freesurfer version 6.0.0 (surfer.nmr.mgh.harvard.edu). Cortical reconstruction, volumetric segmentation and cortical thickness extraction used *recon-all*, and the output was inspected for quality assurance. The individual cortical thickness (CT) maps were then transformed into MNI space using the non-linear mapping method proposed by Wu *et al.* (2018).

The diffusion MRI data were skull-stripped and motion corrected using FSL v5.0.9, and the diffusion tensor model fitted using a non-linear fitting algorithm implemented in C and matlab. Fractional anisotropy (FA) and mean diffusivity (MD) were computed for each subject. FA and MD maps were transformed onto a common template space using DTI-TK, a tensor-based registration approach (Zhang *et al.*, 2006, 2007*a*) and a study-specific population-based atlas (Zhang *et al.*, 2007*b*).

### Feature extraction

For the GM volume and cortical thickness maps, feature extraction was performed in two ways: (i) using the cortical and subcortical regions-of-interest from the Harvard-Oxford Atlas (neuro.imm.dtu.dk/wiki/Harvard-Oxford_Atlas) and (ii) using principal component analysis (PCA).

**Figure 1:**
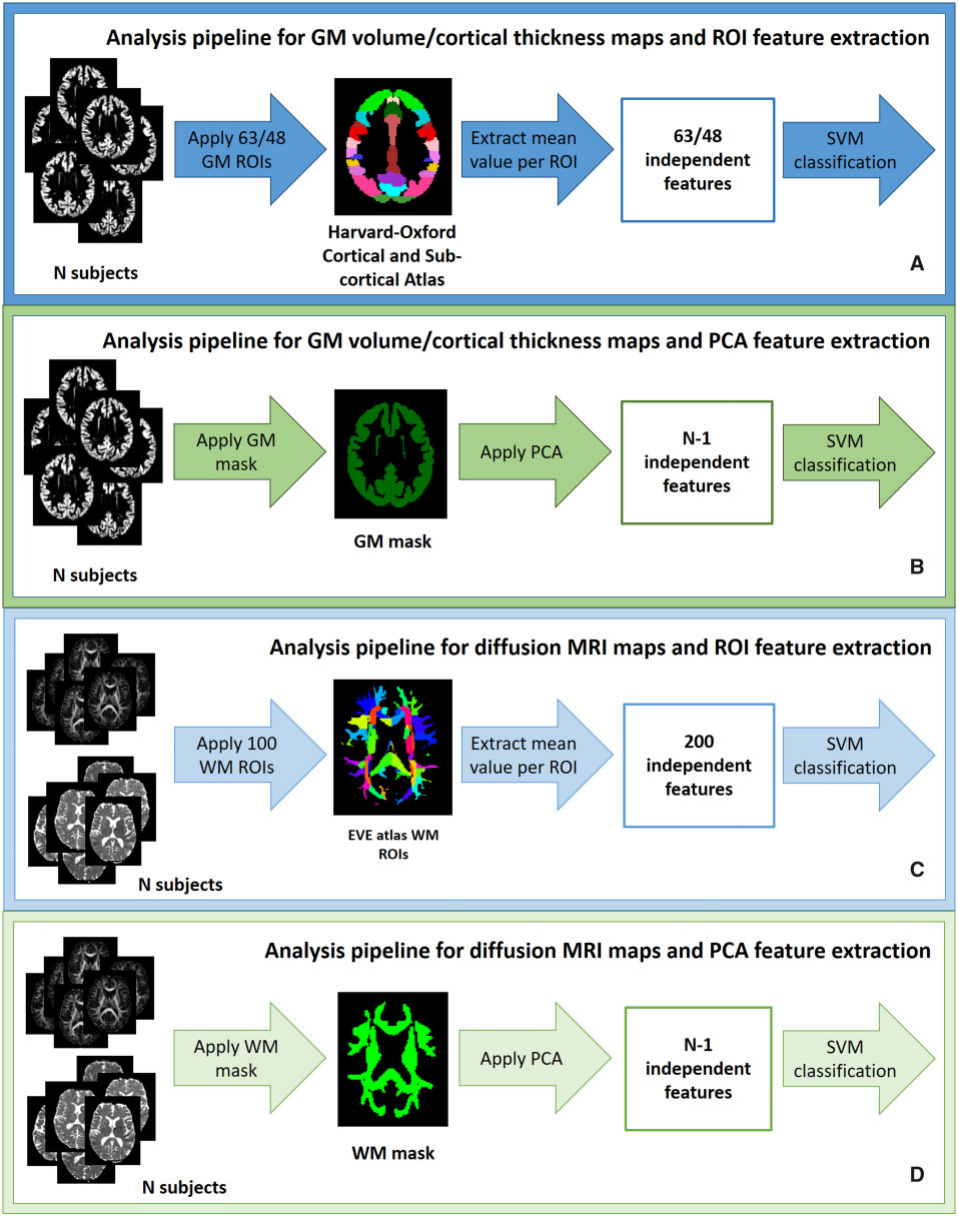
**Data analysis pipelines.** Analysis pipelines for each combination of data type and feature extraction method. (**A**) T1-weighted MRI and ROIs. (**B**) T1-weighted MRI and PCA. (**C**) Diffusion MRI and ROIs. (**D**) Diffusion MRI and PCA.

For the region-of-interest analysis, 63 grey matter cortical and subcortical ROIs were applied to the spatially normalized GM maps for each subject, and the average GM volume value per ROI calculated, hence generating 63 independent features per subject (Fig. 1A). For the PCA analysis, a GM mask was first created by thresholding the mean GM map obtained from DARTEL in MNI space (threshold *P* = 0.1). This mask was applied to the images from each subject, and the voxels contained within the mask were included in a multi-subject PCA analysis, resulting in *N − 1* independent features, where *N* represents the number of subjects included in this analysis (Fig. 1B). Analogous procedures were applied to cortical thickness maps, excluding subcortical ROIs, resulting in 48 cortical-thickness features per subject.

ROI and PCA methods for feature extraction were also applied to the FA and MD maps. For the ROI approach, 100 white matter regions from the EVE atlas (http://lbam.med.jhmi.edu/) were used to extract the average FA and MD values for each region and subject (Fig. 1C). For PCA, a white matter mask was first generated by thresholding the FA map corresponding to the study-specific template (threshold FA ≥ 0.2). Voxels selected from the FA and MD maps of all subjects were included to generate *N − 1* independent features (Fig. 1D).

### Statistical analysis

The subjects included in the cross-validation and independent validation groups were tested for differences in age, UPDRS-III score, MMSE score, TIV and motion metrics using ANOVA, Welch’s ANOVA or Kruskal–Wallis ANOVA depending on whether the relevant assumptions were met for each metric. The assumption of normality was assessed using the following criteria: skewness and kurtosis of residuals between −2 and 2 (George and Mallery, 2010), Shapiro−Wilk test for normality and normal QQ-plots. Homogeneity of variance was accepted if the ratio of the largest residual variance estimate to the smallest group residual variance estimate does not exceed 3 (Dean and Voss, 1999). Analysis of sex differences across groups was performed using Chi-squared testing.

### Feature ranking and statistical classification

Four parallel streams of subsequent analysis were performed, one for each data type and feature extraction method combination: (i) GM maps + ROIs, (ii) GM maps + PCA, (C) diffusion maps + ROIs, and (D) diffusion maps + PCA. Following feature extraction, each feature was individually normalized using its mean and standard deviation (z-scoring). The normalized features generated by each feature extraction approach were then ranked separately, using the Fisher Discriminant Ratio (FDR):

$$\mathrm{FDR}=\frac{{\left(\mu_{1\;}-\mu_{2}\right)}^{2}}{\sigma_{1}^{2}+\sigma_{2}^{2}},$$

where $\mu_{i}\;$and $\sigma_{i}^{2}\;$denote the mean and the variance of the *i*th class, respectively.

The top feature for each stream was used in combination with support vector machines (SVMs) to construct a statistical model for pairwise classification of healthy controls, Parkinson’s disease, CBS and PSP-RS. The remaining features were added to the model, one at a time, in the order of their FDR ranking, and the classification accuracy of each model as a function of the number of features was calculated. The SVM analysis was performed using the LIBSVM package in matlab (Chang and Lin, 2011).

To assess the accuracy of the four analysis streams, we first used leave-*n*-out cross-validation. The *N* available subjects are randomly split into a training set of size *(N* − *n)* and a test set of size *n*. The *N-n* training set is used to build a model, whose performance is tested on the *n* test set. In this study, *n *= 2 for the pairwise comparisons, with the testing set including one subject from each group. Multiple rounds of cross-validation are performed for different permutations of the subjects left out of the training set. We report the average classification accuracy across all iterations of the cross-validation. However, this method may inflate classification accuracies. Therefore the leave-two-out cross-validation was supplemented by an independent validation using a different set of cases altogether. For the cross-validation approach, feature ranking using FDR was recalculated for each fold using the subjects in the training subgroup only, and the same ranking applied to the two subjects left out. For the independent validation, the FDR ranking was determined using the cross-validation group, and the ranking order applied to the independent group.

To facilitate comparison between MRI modalities and feature extraction methods, all feature selection used FDR ranking. We report classification accuracies as a function of the number of ranked features included in the model. Optimal feature selection for each pairwise comparison (group), MRI modality (T1w, DWI) and method (ROI, PCA) might use different numbers of features for each combination. Therefore, we present the accuracies (range, mean and maximum) obtained across the range of features in the classification model. The progressive inclusion of ranked features leads to non-independent observations (the first k features are also included in k + 1 features), so we do not use serial null hypothesis testing of differences between combinations of methods. Instead, we report the range of classification accuracies obtained with each combination of methodologies.

### Spatial localization of the most relevant features

To illustrate the most relevant features that support the SVM classifications between groups (19 controls, 19 Parkinson’s disease, 19 CBS and 19 PSP-RS), we present a map of the SVM weighting for each feature. The parameter weights for each pairwise group-comparison were rescaled so that the most useful feature for each comparison has a normalized weight of 1. We present GM regions from the Harvard-Oxford Atlas to show the most relevant brain features for group discrimination. A similar process was repeated for white matter ROIs in the EVE Atlas, where each ROI was assigned a weight for its FA value and a weight for its MD value. The rescaling was performed for FA and MD features together, so that the top diffusion feature has weight of 1.

### Data availability

Participant consent prevents open data access but academic (non-commercial) requests for data sharing would be welcome. Please contact the senior author. The principal software used (SPM, FSL, LIBSVM and matlab) are publicly available.

## Results

### Quality assurance and subject exclusion

Examples of MRI images for the subjects excluded by the motion quality control assessment are shown in Supplementary material, Section A. Exclusion criteria reduced the sample size to 62 controls (7 subjects excluded by DWI motion metrics), 32 Parkinson’s disease (1 subject excluded by DWI motion metrics, 2 subjects excluded by both DWI and MPRAGE metrics), 33 PSP-RS (16 subjects excluded by DWI motion metrics, 3 subjects excluded by both DWI and MPRAGE metrics) and 26 CBS (6 subjects excluded by DWI motion metrics, 4 subjects excluded by both DWI and MPRAGE metrics).

### Cross-validation and validation groups

After quality assurance, patient groups were confirmed to be matched for motion metrics, total intracranial volume (TIV), age and sex (Table 1). The 62 controls were younger than the patients, and included a larger proportion of females. We therefore randomly removed females and younger subjects to reach a sample of 43 age- and sex-matched healthy controls.

**Table 1:** Demographics, clinical severity scores and quality control information.

	Controls	PD	PSP-RS	CBS	Group difference
Cross-validation group					
Sample size	19	19	19	19	
Sex F/M (%)	36.8/63.2	47.4/52.6	42.1/57.9	52.6/47.4	*P* = 0.786^a^
Age (years)	66.2 ± 1.6 (54.9–81.1)	65.0 ± 1.9 (46.9–76.9)	69.1 ± 1.3 (60.8–83.2)	68.2 ± 2.3 (39.1–88.2)	*P* = 0.373^b^
UPDRS-III score	−	20.5 ± 2.1 (5–32)	27.2 ± 3.4 (8–44)	28.9 ± 3.8 (2–51)	*P* = 0.110^c^
MMSE score	29.1 ± 0.2 (27–30)	29.1 ± 0.3 (26–30)	25.9 ± 0.8 (19–30)	26.7 ± 0.9 (16–30)	*P* < 0.001^d^
TIV (×10^5^)	7.6 ± 0.17 (5.8–9.0)	7.5 ± 0.16 (6.4–8.8)	7.3 ± 0.16 (6.2–8.4)	7.7 ± 0.17 (6.2–8.8)	*P* = 0.274^e^
MPRAGE smoothness FWHM	2005.53 ± 34.7 (1796.92–2275.20)	1993.3 ± 33.0 (1749.62–2296.70)	2088.2 ± 30.3 (1801.92–2364.93)	2047.7 ± 38.8 (1809.02–2401.53)	*P* = 0.202 ^f^
Absolute head displacement (DWI)	1.62 ± 0.09 (1.30–2.77)	1.85 ± 0.17 (1.25–3.82)	1.71 ± 0.11 (1.27–3.38)	1.61 ± 0.08 (1.25–2.43)	*P* = 0.683^g^
Relative head displacement (DWI)	0.51 ± 0.02 (0.26–0.75)	0.48 ± 0.03 (0.13–0.71)	0.42 ± 0.02 (0.28–0.59)	0.44 ± 0.03 (0.18–0.60)	*P* = 0.119^h^
Independent validation group					
Sample size	24	13	14	7	
Sex F/M (%)	58.3/41.7	38.5/61.5	50.0/50.0	42.9/57.1	*P* = 0.655^i^
Age (years)	69.7 ± 1.4 (51.6–81.6)	69.1 ± 1.9 (54.1–75.8)	70.9 ± 1.9 (57.9–84.1)	62.2 ± 3.0 (53.1–75.0)	*P* = 0.064^j^
TIV (×10^5^)	7.4 ± 0.16 (6.0–9.2)	7.5 ± 0.19 (6.1–8.2)	7.7 ± 0.29 (6.7–8.6)	7.3 ± 0.03 (6.0–9.0)	*P* = 0.732^k^
MPRAGE smoothness FWHM	1989.6 ± 24.7 (1781.9–2238.2)	2048.9 ± 46.2 (1824.9–2380.2)	2092.1 ± 28.5 (1937.1–2266.6)	2061.2 ± 78.9 (1863.2–2364.5)	*P* = 0.103^l^
Absolute head displacement (DWI)	1.46 ± 0.04 (1.19–1.98)	1.47 ± 0.08 (1.27–2.44)	1.59 ± 0.06 (1.31–2.01)	1.62 ± 0.11 (1.25–1.96)	*P* = 0.119^m^
Relative head displacement (DWI)	0.48 ± 0.02 (0.23–0.70)	0.48 ± 0.02 (0.25–0.59)	0.44 ± 0.03 (0.32–0.61)	0.48 ± 0.05 (0.30–0.65)	*P* = 0.7827^n^

Data are shown as mean ± standard error (range).

a Chi-squared test,

b ANOVA,

c ANOVA,

d Kruskal–Wallis ANOVA followed by non-parametric Mann–Whitney *post hoc* tests (Control > CBS *P *< 0.01, Control > PSP-RS *P* < 0.05, Parkinson’s disease > CBS *P* < 0.01 and Parkinson’s disease > PSP-RS *P* < 0.05),

e ANOVA,

f ANOVA,

g Kruskal–Wallis ANOVA,

h ANOVA,

i Chi-squared test,

j ANOVA,

k ANOVA,

l Welch’s ANOVA,

m Kruskal–Wallis ANOVA,

n ANOVA.

More details about the statistical results presented here, including justification for choice of statistical test, can be found in the Supplementary material, Section B.

For the cross-validation group, 19 patients were selected with each diagnosis, matching demographics and UPDRS-III, with 19 controls matched for motion metrics, age and sex. The remaining 58 subjects (24 controls, 13 Parkinson’s disease, 14 PSP-RS and 7 CBS) formed the independent validation test cohort.

Table 1 shows the demographic and neuropsychological evaluation scores for all groups. For the cross-validation group, there was no significant difference by diagnosis in sex, age or UPDRS-III score. There was a significant difference in MMSE score across the different groups: *post hoc* tests revealed that both PSP-RS and CBS patients had a lower MMSE compared to healthy controls and Parkinson’s disease patients. Motion metrics were matched across groups. For the independent validation group age and head motion were matched across groups, but there were mild differences between PSP-RS and controls or CBS in terms of age or smoothness respectively (see Table 1). A summary of the motion metrics for datasets excluded after quality control are presented in Supplementary material, Section C.

### Comparison between GM volume and cortical thickness

**Figure 2:**
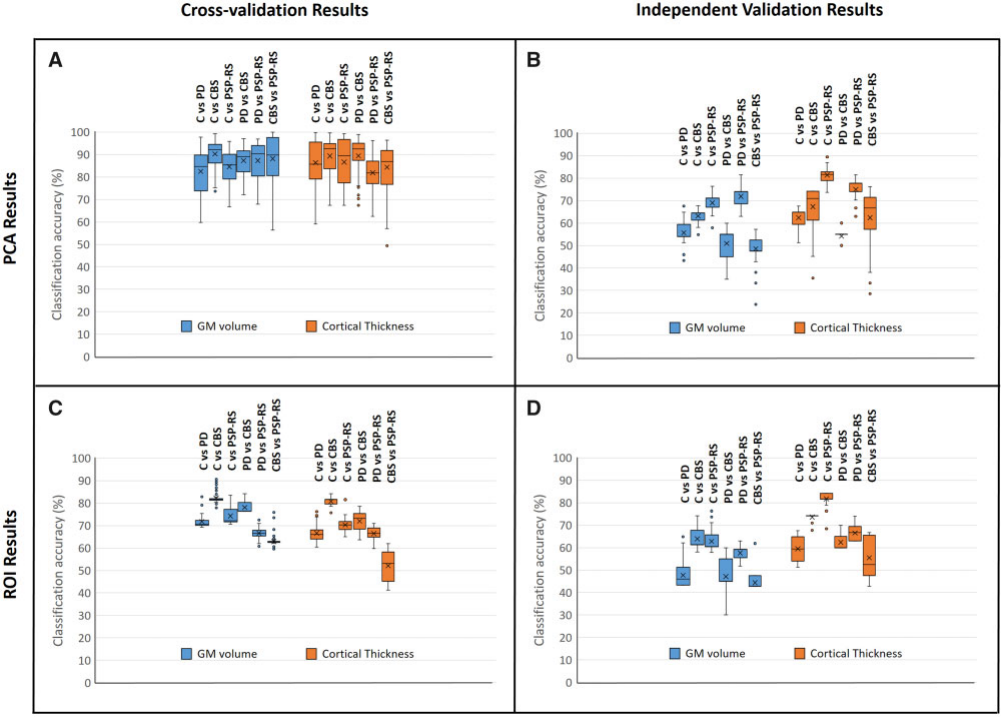
**GM volume versus cortical thickness.** Comparison between GM volume (blue) and cortical thickness (orange) as feature types. The range of classification accuracies presented for each pairwise comparison and combination of methodological variables corresponds to the results obtained as different numbers of features are included in the statistical model. (**A**) Cross-validation results when PCA is used for feature extraction. (**B**) Independent validation results when PCA is used for feature extraction. (**C**) Cross-validation results when ROIs are used for feature extraction. (**D**) Independent validation results when ROIs are used for feature extraction.

Figure 2 shows the classification results using GM volume or cortical thickness. The methods performed similarly when cross-validation is used with PCA features (Fig. 2A). However, using ROI features, cortical thickness underperforms relative to GM volume, for all pairwise comparisons (Fig. 2C). The reverse is observed for the independent validation results where classification accuracies are lower for GM volume features, for all pairwise comparisons (Fig. 2B and D). The performances of different ROI atlases for thickness feature extraction are presented in Supplementary material, Section D. Since the results of GM volume and thickness are similar, we focus the remaining analyses on GM volume features as used in most previous classification studies.

### Cross-validation results

Cross-validation classification results are presented in Table 2, for both GM volume and diffusion data. The mean and maximum accuracies and IQR were calculated over the number of features used (ROIs or PCA components). The accuracy results for group comparisons are all above chance (50%), but some are lower than previous reports from unmatched studies (e.g. Haller *et al.*, 2012; Salvatore *et al.*, 2014). With the well-matched groups described in our study, mean classification accuracies were in the range 62.16–90.3% for T1-weighted data and 61.26–94.95% for diffusion data.

**Table 2:** Classification accuracies achieved for pairwise comparisons using a leave-two-out cross-validation approach

	Mean accuracy (%)	IQR (%)	Max accuracy (%)
T1-weighted data (GM volume maps)			
GM maps + ROIs			
C vs PD	71.96	2.18	85.46
C vs CBS	83.36	0.59	91.69
C vs PSP-RS	73.74	5.68	81.02
PD vs CBS	77.93	3.88	85.87
PD vs PSP-RS	67.44	2.35	70.91
PSP vs CBS	62.16	0.55	65.65
GM maps + PCA			
C vs PD	82.54	15.17	97.78
C vs CBS	90.33	8.00	99.31
C vs PSP-RS	84.60	10.77	95.84
PD vs CBS	87.31	9.28	97.09
PD vs PSP-RS	87.38	13.50	96.95
PSP vs CBS	88.23	16.17	100.0
Diffusion MRI data (FA and MD maps)			
FA and MD maps + ROIs			
C vs PD	61.26	12.88	75.21
C vs CBS	70.13	5.54	77.28
C vs PSP-RS	82.44	6.44	87.53
PD vs CBS	72.89	10.66	81.99
PD vs PSP-RS	74.93	6.58	85.04
PSP vs CBS	79.84	7.89	90.72
FA and MD maps + PCA			
C vs PD	85.43	19.43	99.72
C vs CBS	90.06	9.90	97.37
C vs PSP-RS	92.51	10.15	99.86
PD vs CBS	84.40	7.34	91.55
PD vs PSP-RS	89.49	3.98	96.40
PSP vs CBS	94.95	12.67	100.0

For each pairwise comparison, two patients, one from each group, were left out of the training phase for each cross-validation fold and used to estimate model accuracy. The classification accuracies presented correspond to the mean and maximum accuracies obtained when different numbers of features (ROIs or PCA components) are included in the statistical model. Inter-quartile range (IQR) is also shown.

Plots showing classification accuracy, sensitivity and specificity as a function of the number of features included in the model (number of ROIs or PCA components) can be found in Supplementary material, Section E. Comparisons between controls and PSP-RS and between Parkinson’s disease and PSP-RS are shown as representative examples.

### Independent validation results

Table 3 summarizes the results using independent validation samples to test model accuracy, for both GM volume and diffusion data. The mean and maximum accuracies and IQR were calculated over the number of features used (ROIs or PCA components). Training and testing in less well-matched independent sets of subjects resulted in mean classification accuracies in the range 44.37–71.87% for T1-weighted data and 57.63–90.49% for diffusion data.

**Table 3:** Classification accuracies achieved using the independent validation group

	Mean accuracy (%)	IQR (%)	Max accuracy (%)
T1-weighted data (GM volume maps)			
GM maps + ROIs			
C vs PD	47.75	8.11	64.86
C vs CBS	63.95	6.45	74.19
C vs PSP-RS	62.78	4.61	76.32
PD vs CBS	47.14	8.75	60.00
PD vs PSP-RS	57.67	3.70	62.96
PSP vs CBS	44.37	4.76	61.90
GM maps + PCA			
C vs PD	55.66	5.41	67.57
C vs CBS	63.12	3.23	67.74
C vs PSP-RS	68.99	3.29	76.32
PD vs CBS	50.95	10.00	60.00
PD vs PSP-RS	71.87	4.63	81.48
PSP vs CBS	48.52	4.76	57.14
Diffusion MRI data (FA and MD maps)			
FA and MD maps + ROIs			
C vs PD	59.74	5.41	75.68
C vs CBS	78.74	5.37	87.09
C vs PSP-RS	90.49	2.63	94.74
PD vs CBS	77.90	15.00	85.00
PD vs PSP-RS	86.78	3.70	92.59
PSP vs CBS	76.33	4.76	80.95
FA and MD maps + PCA			
C vs PD	57.63	10.81	72.97
C vs CBS	73.41	6.45	80.64
C vs PSP-RS	80.87	5.92	89.44
PD vs CBS	80.81	5.00	85.00
PD vs PSP-RS	81.48	3.70	88.89
PSP vs CBS	80.82	4.95	90.63

Seventy subjects (19 from each group) were used to train the model, and validation was performed on 58 unseen patients and controls. The classification accuracies presented correspond to the mean and maximum accuracies obtained when different numbers of features are included in the statistical model (ROIs or PCA components). Inter-quartile range (IQR) is also shown.

Plots showing classification accuracy, sensitivity and specificity as a function of the number of features included in the model (number of ROIs or PCA principal components) can be found in Supplementary material, Section F. Comparisons between controls and PSP-RS and between Parkinson’s disease and PSP-RS are shown as representative examples.

### Comparison between feature extraction methods (ROIs versus PCA)

**Figure 3:**
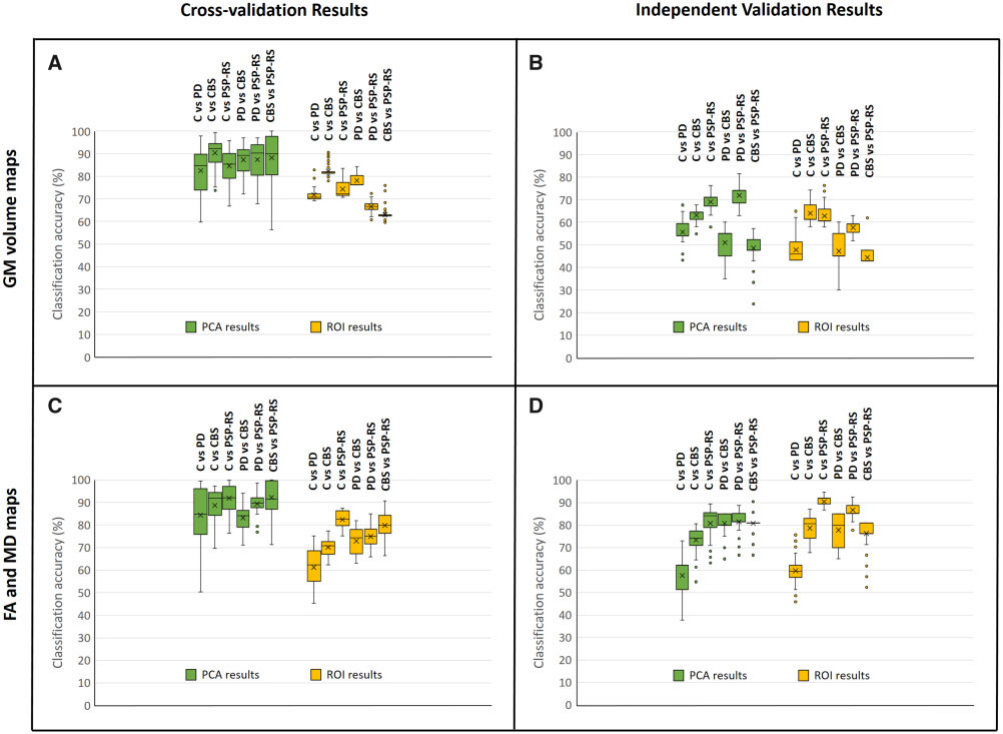
**PCA versus ROIs.** Comparison between feature extraction methods: PCA (green) and ROIs (yellow). The range of classification accuracies presented for each pairwise comparison and combination of methodological variables corresponds to the results obtained as different numbers of features are included in the statistical model. (**A**) Cross-validation results when GM volume maps are used as feature type. (**B**) Independent validation results when GM volume maps are used as feature type. (**C**) Cross-validation results when FA and MD maps are used as feature type. (**D**) Independent validation results when FA and MD maps are used as feature type.

Figure 3 shows the classification accuracies for each method of feature extraction, across all pairwise group comparisons, for cross-validation and independent validation. Note the comparison between data reduction methods (PCA green, ROI yellow) and feature types (GM volume top row, FA+MD bottom row), for within sample cross-validation (left) and out-of-sample independent validation (right).

### Comparison between cross-validation and independent validation

**Figure 4:**
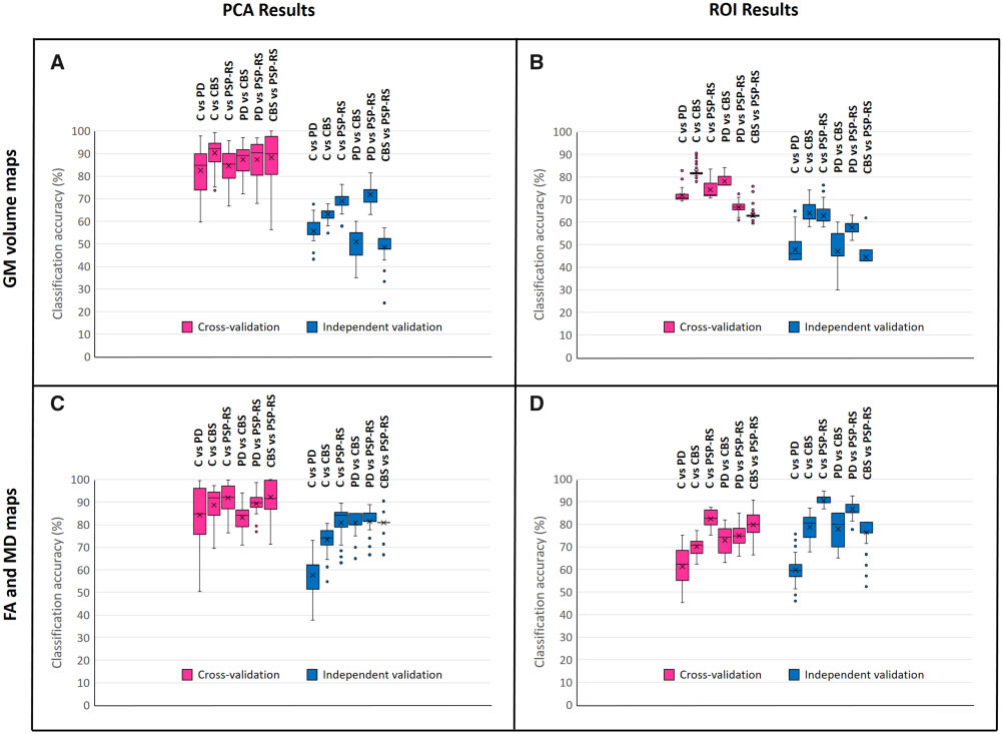
**Cross-validation versus independent validation.** Comparison between cross-validation (magenta) and independent validation (blue) results. The range of classification accuracies presented for each pairwise comparison and combination of methodological variables corresponds to the results obtained as different numbers of features are included in the statistical model. (**A**) Results obtained when PCA is used for feature extraction, with GM volumes maps as feature type. (**B**) Results obtained when ROIs are used for feature extraction, with GM volumes maps as feature type. (**C**) Results obtained when PCA is used for feature extraction, with FA and MD maps as feature type. (**B**) Results obtained when ROIs are used for feature extraction, with FA and MD maps as feature type.

Only qualitative descriptions are used to compare between the two methods for model validation (see Methods section). Figure 4 shows box plots for the range of classification accuracies obtained with each approach, across all pairwise comparisons, for both types of feature extraction (ROIs and PCA components).

### Spatial localization of the most relevant features

**Figure 5:**
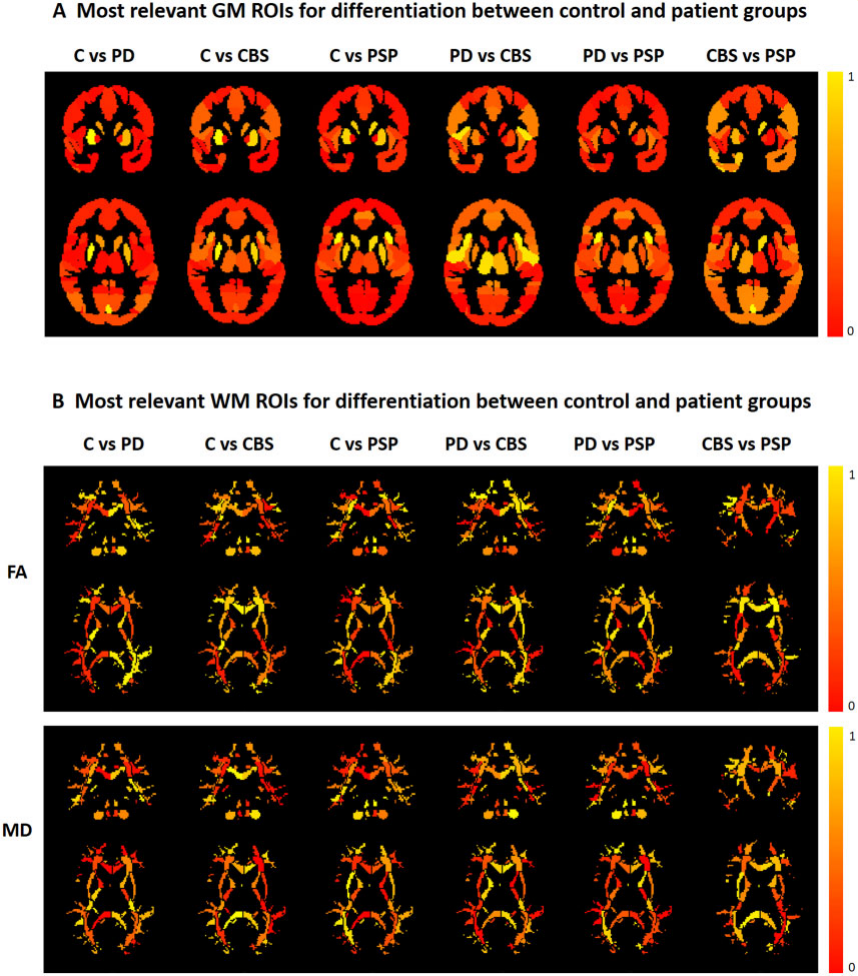
**Spatial localization of top classification features.** Colour-coded images showing the relative importance of each ROI for pairwise classification using SVM. The top ranked feature for each pairwise group comparison has a normalized weight of 1 and is shown in yellow, while the least important features are shown in red. (**A**) Cortical and sub-cortical ROIs used for classification with GM volume features. (**B**) White matter ROIs used for classification with FA and MD features. The top row shows the spatial distribution of the most relevant FA features, while the bottom row shows the localization of the most relevant MD features.

Figure 5A indicates the relative importance of each GM ROI for classification (the model used for independent validation). Figure 5B shows the analogous maps of diffusion features. These are the most relevant regional features for the classification accuracies summarized in Table 3.

## Discussion

This study addresses four key issues in the use of MRI for diagnostic or classification biomarkers for parkinsonian disorders. We show that even with well-matched groups, of equal size, and tight control of motion artefacts, one can achieve accurate cross-validated differential diagnosis of Parkinson’s disease, PSP-RS and CBS. The strongest results were achieved when principal components analysis was used for feature extraction, resulting in consistent mean accuracies above 80% and maximum accuracies over 90% for all group comparisons, using either diffusion or grey matter volume data. The use of PCA feature extraction gave higher accuracies than ROIs, with a difference of approximately 25%. While mapping the distribution of pathology was not an aim of the present study, the regions that were most contributory to the support vector machine classifiers were biologically plausible, including the basal ganglia. Using diffusion weighted images for classification, the highly ranked features included diffuse FA and MD signals, and neither metric outperformed the other (FA versus MD).

Overall, we confirmed that good diagnostic accuracy can be achieved using either grey or white matter features from standard structural and diffusion MRI sequences, respectively. The classifications by diffusion and GM volume data were generally similar for the cross-validation group (‘within-sample’). However, there were some notable differences. For example, diffusion data resulted in better differentiation than volume data between PSP-RS and CBS patients (80% versus 62%), but poorer differentiation between controls and Parkinson’s disease (61% versus 72%). Neither data type outperformed the other in all cases, although diffusion data produced higher accuracies when comparing PSP-RS patients to controls or CBS patients. With the independent validation groups, accuracy generally decreased (by an average 13%), despite less stringent matching. This decrease may indicate overestimation by within-sample cross-validation. However, the difference was less marked when diffusion metrics were used (5% average reduction in accuracy) than GM volumes (average 23% reduction in accuracy, some to chance levels). Using a principal components analysis over grey matter volume of white matter diffusion signals provides higher classification accuracies compared to a set of anatomical regions-of-interest when cross-validation is used to estimate accuracy, but lower classification accuracies in the independent validation analysis.

The selection of imaging features is critical to the performance and interpretation of classifiers. MRI provides a rich repertoire of structural, functional, neurochemical and diffusion features. We focus on the T1-weighted and diffusion tensor images which are most widely available, with short sequences that are readily tolerated by patients, and which require minimal operator expertise. These would be an advantage for scalable multisite studies, or in support of diagnostics and stratification in a trial context. Nonetheless, even these standard sequences provide many potential features and feature extraction options.

We compared two approaches for feature extraction, based on (i) *a priori* regions of interest from a common anatomical atlas and (ii) a data-driven principal components analysis. When PCA was used for feature extraction, accuracy, sensitivity and specificity generally increase as more features are added to the model until a plateau at ∼15 components for GM volume data and 15-30 for diffusion data (see the plots in Supplementary material, Section E). The plateau is generally sustained until the last 2–5 features are added, consistent with previous studies (Salvatore *et al.*, 2014). This is expected for PCA with FDR feature-ranking, which concentrate predictive information in the earlier features. When ROIs are used as features, only the FDR criterion is used for feature ranking and the ranking of features may differ for each fold of the cross-validation. The non-predictive information remains more evenly distributed across ROI features than PCA features. The advantage of PCA could be due to small localized changes in brain morphology and/or function that are averaged across a ROI. On the other hand, the differences between the two feature extraction methods are significantly reduced when an independent sample is used for validation. This suggests that the PCA approach may be more vulnerable to the overfitting with cross-validation approaches.

We also compared two types of tissue signal—GM volume and thickness measures based on a T1-weighted sequence, and metrics of white matter tissue organisation using diffusion tensor imaging. We replicated previous studies in that both types of data support classification above chance. Neither type clearly outperforms the other across all pairwise comparisons among our three clinical cross-validation groups. However, features obtained from diffusion MRI data resulted in higher classification accuracies using the independent validation cohort (for both ROIs and PCA feature extraction). For some key contrasts of interest (Parkinson’s disease versus CBS, and Parkinson’s disease versus PSP-RS) the classification accuracy in the independent sample using diffusion data was as good as the cross-validation results. Despite the improvement of thickness over volume in the independent validation, diffusion metrics still outperformed GM thickness for three out of the six groupwise comparisons (Supplementary material, Section G). In the main results, we combined FA and MD features for the classifier, although single diffusion metrics [i.e. FA-only or MD-only were not consistently inferior (Supplementary material, Section H)]. Interestingly, the combination of diffusion and GM volume features did not increase classification accuracy with FDR feature ranking (Supplementary material, Section I).

Close matching by demographics, clinical severity and motion artefacts is essential to properly evaluate and compare candidate biomarkers. Without such matching, the apparent success of some previous imaging-based biomarkers in distinguishing clinical groups may have been inflated by individual differences that are unrelated to the structural and neuropathological consequences of disease. For example, in unselected cases, motion artefacts were greater in patients than controls: 26% of patients exceeded our motion criteria compared to only 10% of controls. Exclusion rates varied by group: 9% of Parkinson’s disease, 28% of CBS and 37% of PSP-RS.

Machine learning tools such as support vector machines are very sensitive to systematic patterns in the data but are agnostic as to the origins of such patterns e.g. motion versus neuropathology versus atrophy. The very high classification accuracies between patient groups reported in some previous studies (up to 100%), may be inflated by differential motion. The effects of head motion in MRI data analysis are well documented. For example, head motion during acquisition of 3D T1-weighted MRI images reduces grey matter volume estimates (Reuter *et al.*, 2015), while head motion in a diffusion MRI acquisition can create spurious group differences (Yendiki *et al.*, 2014). Similarly, the comparison of groups at different stages of disease, or different levels of severity, may confound classification. Unfortunately, there is no universal severity or staging rating scale across parkinsonian disorders, with disease-specific features in the UPDRS and PSP-rating-scale. The forthcoming PSP functional rating scale and new CBS functional rating scale may be applicable across groups, but we applied the available UPDRS-III with its focus on common motor features across our three clinical groups.

Despite matching by age, sex, motion artefacts, and having similar UPDRS-III scores, the issue of differential disease severity remains challenging, from two perspectives. First, there is currently no single rating scale or investigation that fully summarizes disease severity across Parkinson’s disease, PSP and CBS, either as a clinical scale, neurotransmitter or functional brain image. Even where a clinical scale such as UPDRS is applicable across the disorders, it may not give a like-for-like index of disease stage (e.g. from onset to death) or functional decline (e.g. activities of daily living), or pathology (e.g. dopamine depletion, or cell loss). Second, the three diseases may each have prolonged prodromal phases and long periods in which patients are misdiagnosed. PSP and CBD typically take 2–3 years from symptoms to diagnosis (Coyle-Gilchrist *et al.*, 2016; Mamarabadi *et al.*, 2018), while Parkinson’s disease causes under-recognized clinical manifestations like constipation and REM-sleep behavioural disorder many years before tremor and akinesia. It is too soon to know whether MRI based classification is capable of differentiating these disorders in early prodromal stages, or pre-symptomatically, in the way that has been shown for frontotemporal dementia (Rohrer *et al.*, 2015). For PSP, the recent operationalization of early stage ‘oligosymptomatic’ and ‘possible’ cases will enable MRI biomarkers of PSP to be tested earlier (Höglinger *et al.*, 2017).

Phenotypic variation other than severity is also challenging. The classical Richardson’s syndrome presentation of PSP has very high clinico-pathological correlations to PSP-pathology. However, this classical phenotype may represent a minority of presentations of PSP-pathology: cognitive, linguistic and behavioural presentations are common (Respondek *et al.*, 2014; Höglinger *et al.*, 2017). Similarly, CBS has many phenotypic variants, with motoric, behavioural and language presentations (Armstrong *et al.*, 2013). This study does not include cases from the full phenotypic range of corticobasal syndromes, or syndromes caused by corticobasal degeneration (Alexander *et al.*, 2014). The current study was not designed to resolve the issue of heterogeneity, but rather to highlight methodological considerations, and best practice, which we hope can be carried forward to identify robust biomarkers of a wide range of phenotypic expressions of the pathologies of Parkinson’s disease, PSP and corticobasal degeneration (Jabbari *et al.*, 2020).

Although we have addressed four key methodological issues, several limitations remain. This was a single centre study, resulting in a modest sample size when compared to recent multi-centre studies (Huppertz *et al.*, 2016; Nigro *et al.*, 2017; Jabbari *et al.*, 2020). This limits the generalisation of our results to different clinical sites with potentially different scanning practices, scanner manufacturers and sequence parameters. Our diffusion data were not corrected for EPI distortions, as data were acquired before reverse-phase encode direction acquisitions was common practice. Therefore, the FA and MD maps used for classification were affected by distortion artefacts, albeit in all groups. The control and patient groups included in this study were matched for age, sex, motion parameters and UPDRS-III scores (for the patients). However, there was a difference in MMSE between the groups; and previous studies have highlighted the cognitive impairments resulting from Parkinson’s disease (Williams-Gray *et al.*, 2013), PSP (Rittman *et al.*, 2013) and CBS (Burrell *et al.*, 2014). Given the correlations between cognitive function and structural and diffusion MRI in Parkinson’s disease, PSP-Richardson’s syndrome, and CBS (Paviour *et al.*, 2006; Ghosh *et al.*, 2012; Rae *et al.*, 2012; Mak *et al.*, 2015) the non-matching by cognitive dysfunction could contribute to classification. Against this argument, is that different cognitive deficits are hallmarks of Parkinson’s disease, PSP and CBS, and to match a cognitive profile would compromise the representativeness of the patients chosen. Another potential limitation of this study is that patient labels were assigned using clinical diagnostic criteria not histopathology, and the limitation of clinicopathological correlations may cap the statistical classifier’s ability to learn and separate the different patterns of disease. Our centre’s diagnostic accuracy of CBS and PSP-RS is in line with other centres (Alexander *et al.*, 2014; Gazzina *et al.*, 2019), with generally high clinicopathological correlation of PSP-Richardson’s syndrome (>90%) relative to CBS/CBD (>60%). Finally, all our data were subjected to strict data quality control criteria, with the aim that the disease patterns detected by SVM were independent of the severity of motion present in those data. While this ensures that poor data quality will not be mistaken for real effects of the pathology, it may also exclude patients with symptoms that do not allow them to be still enough to undergo the MRI examination. For example, 19 subjects with PSP-RS (37% of the original sample) were excluded by our quality control criteria, which may bias the sample in the PSP-RS group. This indicates a tradeoff, whereby high-quality data cohorts may not be representative of the full range of disease.

In summary, we suggest that machine learning methods for MRI data can be used to aid the automatic differential diagnosis of PSP-RS, CBS and Parkinson’s disease, meeting critical criteria set by the Movement Disorder Society Neuroimaging Study Group and the JPND Working group ASAP-SYn-Tau (van Eimeren *et al.*, 2019). However, to make such a contribution, and augment clinical assessments, these techniques must guard against methodological biases from different levels of motion across patient groups, and poorly matched samples. With closely matched groups, of equal size and similar severity, the use of diffusion weighted images is particularly encouraging, in its high accuracy rate and generalization to independent data. Application of these methods to large samples and multisite studies will be facilitated by international collaborative studies of early stage or atypical presentations of each disease [e.g. PROSPECT-UK (Woodside *et al.*, 2017) and the Four-repeat tauopathy neuroimaging initiative], aiming for reliable, unbiased, disseminated tools for early differential diagnosis and stratification in clinical trials of new therapies.

## Funding

This work was supported by the Medical Research Council (SUAG/051 G101400, SUAG/058 G101400); the Wellcome Trust (103838); the Guarantors of Brain; the Raymond and Beverley Sackler Trust; Lundbeck Fonden; the National Institute for Health Research Cambridge Biomedical Research Centre and the Cambridge Centre for Parkinson-Plus.

## Competing interests

The authors declare no competing interests.

## Supplementary Material

fcaa051_Supplementary_Data

## Abbreviations

3D =: three dimensional
ASAP-SYn-Tau =: alignment and standardization of neuroimaging methods in atypical Parkinsonism, specifically synucleinopathies and tauopathies
C =: C programming language
CBD =: corticobasal degeneration
CBS =: corticobasal syndrome
CT =: cortical thickness
DARTEL =: diffeomorphic anatomical registration through exponentiated lie algebra
DTI =: diffusion tensor imaging
DTI-TK =: diffusion tensor imaging tool kit
DWI =: diffusion weighted imaging
FA =: fractional anisotropy
FDR =: Fisher discriminant ratio
SL =: FMRIB Software Library
FoV =: field of view
GM =: grey matter
GRAPPA =: generalized autocalibrating partially parallel acquisition
IQR =: inter-quartile range
JPND =: EU Joint Programme Neurodegenerative Disease
LIBSVM =: Library for Support Vector Machines
MD =: mean diffusivity
MDS =: Movement Disorder Society
MMSE =: mini mental state examination
MNI =: Montreal Neurological Institute
MPRAGE =: magnetization prepared rapid gradient echo
PAT =: parallel acquisition techniques
PCA =: principal component analysis
PD =: Parkinson’s disease
PROSPECT-UK =: UK-based study of PSP, CBD, MSA and atypical parkinsonism syndromes
PSP =: progressive supranuclear palsy
PSP-RS =: progressive supranuclear palsy Richardson’s syndrome
REM =: rapid eye movements
ROI =: region of interest
SPM12 =: Statistical Parametric Mapping, version 12
SVM =: support vector machines
T1w =: T1 weighted
TBSS =: tract based spatial statistics
TE =: echo time
TIV =: total intracranial volume
TR =: repetition time
TRSE =: twice refocused spin echo
UK =: United Kingdom
UPDRS =: Unified Parkinson’s Disease Rating Scale
VBM =: voxel-based morphometry
WM =: white matter
