# Supplementary material for: Towards accurate and unbiased imaging-based differentiation of Parkinson’s disease, progressive supranuclear palsy and corticobasal syndrome
Source: Brain Commun. 2020 Apr 27;2(1):fcaa051. doi: 10.1093/braincomms/fcaa051 (PMC7325838; doi:10.1093/braincomms/fcaa051)
Supplement: fcaa051_Supplementary_Data [file fcaa051_supplementary_data.pdf]

Supplementary Material

Section A – Examples of datasets excluded by quality control

Below are some examples of MRI images for subjects excluded by the motion quality control procedures.

(A) T1-weighted MPRAGE for a PSP-RS patient identified as outlier by the estimated smoothness of the segmented soft tissue outside the brain.

(B) Two consecutive slices for the diffusion MRI data for a PSP-RS patient identified as an outlier by the absolute and relative displacement metrics.

(C) Diffusion MRI data for a CBS patient identified by the automated stripe detection algorithm.

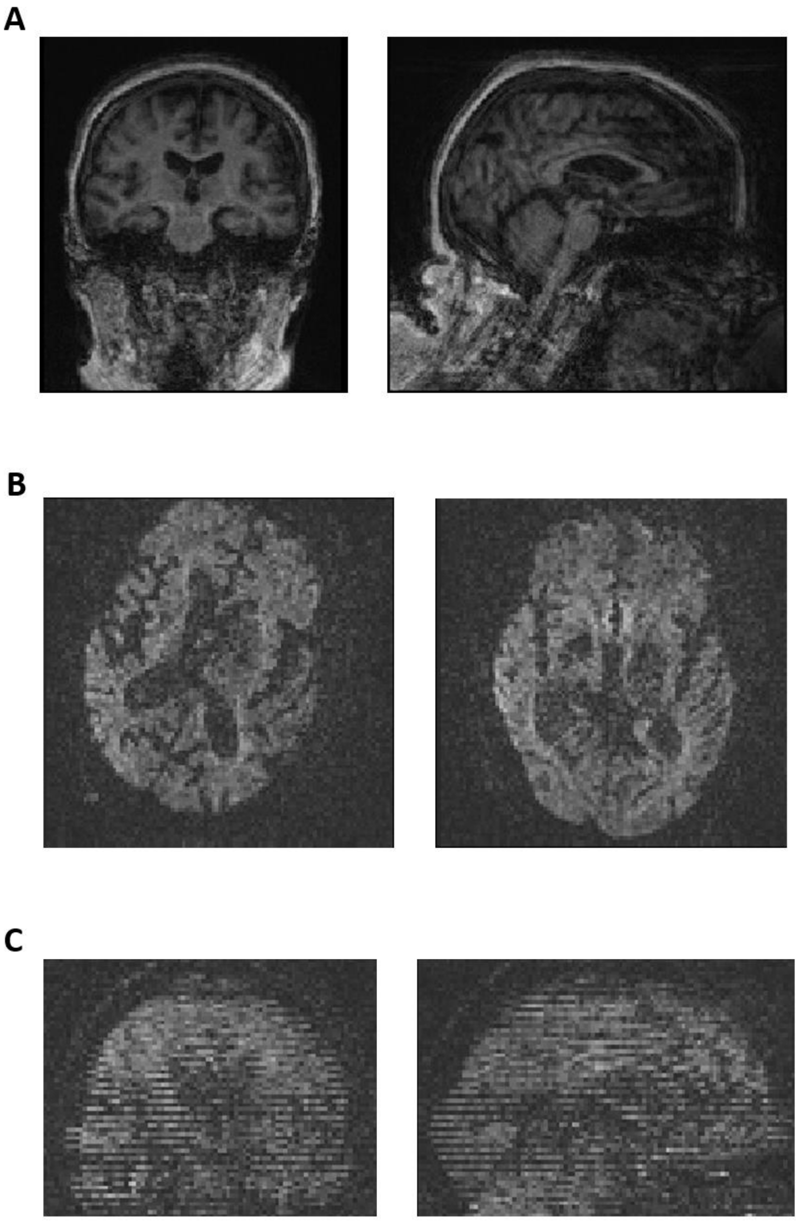

Section B – Details for the statistical test results reported in Table 1

When required, the assumptions of normality and homogeneity of variance were assessed. On normality, we used the following assessment criteria: skewness and kurtosis of residuals between -2 and 2 (George and Mallery, 2010), Shapiro-Wilk test for normality and Normal QQ-plots. Homogeneity of variance was accepted if the ratio of the largest residual variance estimate to the smallest group residual variance estimate does not exceed 3 (Dean and Voss, 1999).

When both assumptions (normality and homogeneity of variance) were valid, an ANOVA test was performed. If the assumption of normality was valid, but the group residual variances were found to be different, the Welch's ANOVA test was performed. If neither assumption was valid, the non-parametric Kruskal-Wallis ANOVA test was performed.

Cross-validation group

a. Sex ratio: Chi-squared test, X^2=1.064, df=3, p=0.786.

b. Age: ANOVA, F=1.057, df=3,72, p=0.373. Shapiro-Wilk test for normality, W=0.970, p=0.066.

Groups	*Average*	*Variance*
C	66.1981255	45.8224841
PD	64.9798125	70.1735278
PSP-RS	69.0889689	34.0031549
CBS	68.2103819	101.463376

ANOVA

	SS	*df*	*MS*	*F*	*P-value*	*F crit*
Between Groups	199.4245	3	66.4748201	1.05741108	0.372649	2.73180701
Within Groups	4526.326	72	62.8656358			
Total	4725.75	75				

	Residuals
Mean	-6.1E-15
Standard Error	0.891119
Median	0.389834
Standard Deviation	7.768591
Sample Variance	60.35101
Kurtosis	1.948453
Skewness	-0.60276
Range	49.05753
Maximum	19.95126
Minimum	-29.1063

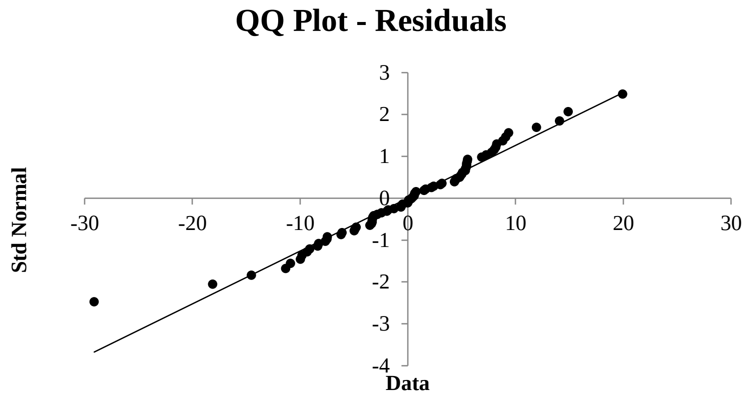

c. UPDRS-III score: ANOVA, F=3.199, df=2,46, p=0.110. Shapiro-Wilk test for normality, W=0.968, p=0.212.

Groups	*Average*	*Variance*
PD	20.47368	85.26316
CBS	28.9375	227.1292
PSP-RS	27.21429	157.4121

ANOVA

	SS	*df*	*MS*	*F*	*P-value*	*F crit*
Between Groups	704.6624	2	352.3312	2.319285	0.109738	3.199582
Within Groups	6988.031	46	151.9137			
Total	7692.694	48				

	Residuals
Mean	0.0000
Standard Error	1.723688435
Median	-0.214285714
Standard Deviation	12.06581905
Sample Variance	145.5839893
Kurtosis	-0.914406001
Skewness	-0.151849567
Range	49
Maximum	22.0625
Minimum	-26.9375

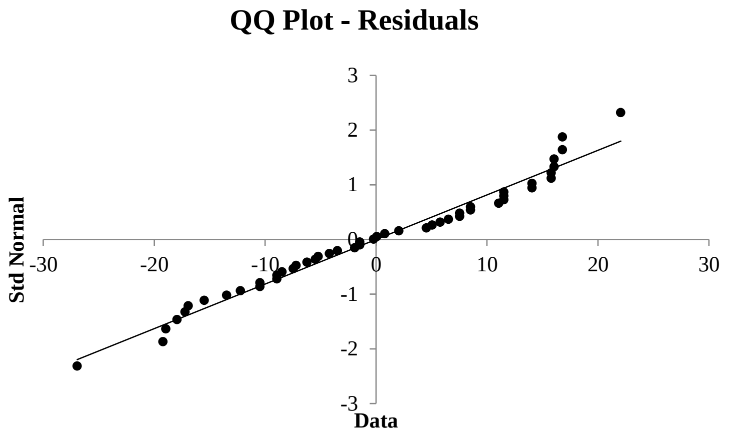

d. MMSE score: Kruskal-Wallis ANOVA, H=17.401, df=3, p<0.001. Shapiro-Wilk test for normality, W=0.884, p<0.001.

Groups	*Average*	*Variance*
C	29.11111	1.045752
PD	29.05263	1.274854
CBS	25.94444	11.23203
PSP-RS	26.70588	14.22059

Kruskal-Wallis Test

	C	PD	CBS	PSP-RS	
Median	29.5	29	27	28	
Rank sum	833.5	871.5	427.5	495.5	
H-stat					16.53768
H-ties					17.40078
df					3
p-value					0.000585

Pairwise Mann-Whitney tests

group 1	*group 2*	*p-value*	*mean*
C	PD	0.935345	0.05848
C	CBS	0.001302	3.166667
C	PSP-RS	0.013158	2.405229
PD	CBS	0.001415	3.108187
PD	PSP-RS	0.010879	2.346749
CBS	PSP-RS	0.384148	0.761438

	Residuals
Mean	-1.5E-16
Standard Error	0.299713
Median	0.888889
Standard Deviation	2.543146
Sample Variance	6.467592
Kurtosis	4.208505
Skewness	-1.56033
Range	14.76144
Maximum	4.055556
Minimum	-10.7059

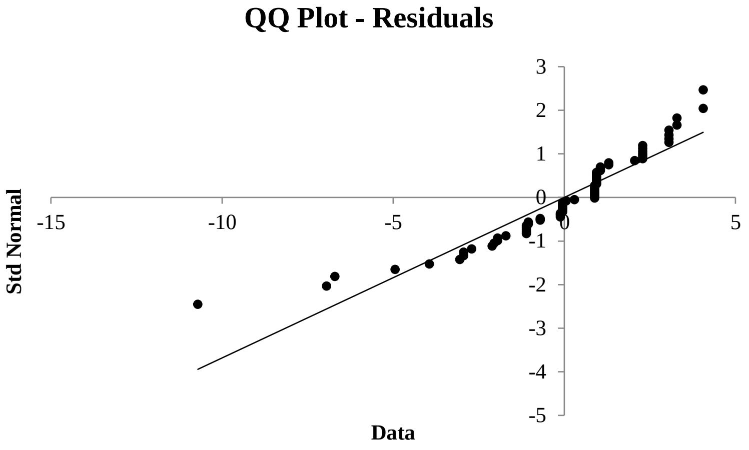

e. TIV: ANOVA, F=1.320, df=3,72, p=0.274. Shapiro-Wilk test for normality, W=0.980, p=0.280.

Groups	*Average*	*Variance*
C	764080.45	5538017372
PD	754308.74	4925220122
CBS	728319.74	4958160310
PSP-RS	772208.58	5502499820

ANOVA						
	SS	*df*	*MS*	*F*	*P-value*	*F crit*
Between Groups	2.0722E+10	3	6907201633	1.32044264	0.274412	2.731807
Within Groups	3.7663E+11	72	5230974406			
Total	3.9735E+11	75				

	Residuals
Mean	0.0000
Standard Error	8128.6817
Median	6672.0338
Standard Deviation	70864.20415
Sample Variance	5021735430
Kurtosis	-0.459630771
Skewness	-0.241441727
Range	328949.1920
Maximum	140446.2073
Minimum	-188502.9847

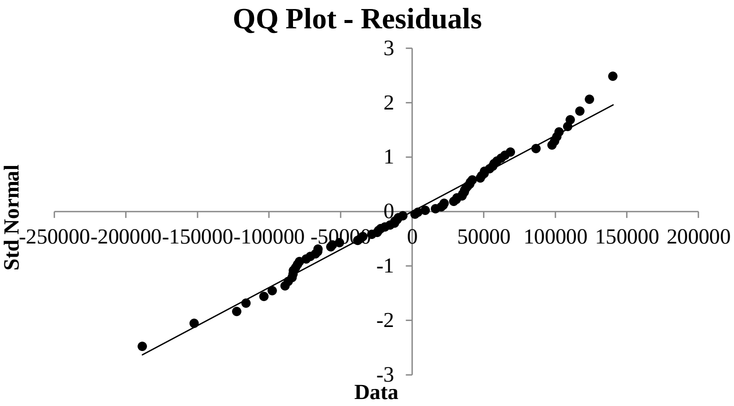

f. <u>MPRAGE FWHM</u>: ANOVA, F=1.579, df=3,72, p=0.202. Shapiro-Wilk test for normality, W=0.981, p=0.299.

Groups	*Average*	*Variance*
CBS	2047.663	28652.36
PSP-RS	2088.163	17425.6
PD	1993.266	20643.39
C	2005.534	22914.29

ANOVA						
	SS	*df*	*MS*	*F*	*P-value*	*F crit*
Between Groups	106199.6	3	35399.86	1.579723	0.201727	2.731807
Within Groups	1613442	72	22408.91			
Total	1719641	75				

	Residuals
Mean	-1.79506E-13
Standard Error	17.98059836
Median	-6.545736108
Standard Deviation	156.7512223
Sample Variance	24570.94571
Kurtosis	-0.553857141
Skewness	0.23462213
Range	641.1836761
Maximum	332.9823716
Minimum	-308.2013045

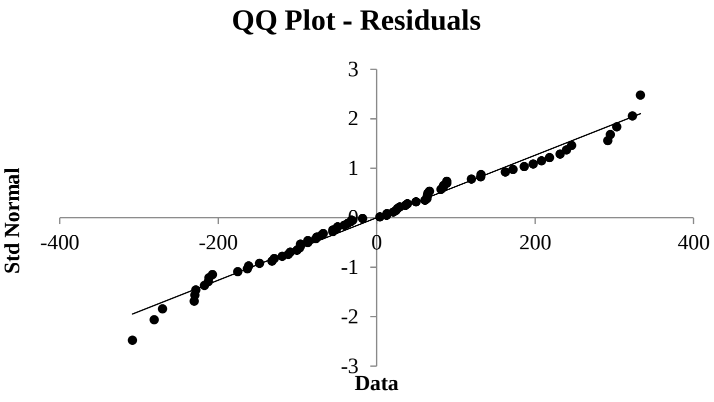

g. Absolute head displacement (DWI): Kruskal-Wallis ANOVA, H=1.496, df=3, p=0.683. Shapiro-Wilk test for normality, W=0.753, p<0.001.

Groups	*Average*	*Variance*
C	1.623796	0.143842
CBS	1.611407	0.11049
PD	1.847374	0.524819
PSP-RS	1.710735	0.222011

Kruskal-Wallis Test

	C	*CBS*	*PD*	*PSP-RS*	
Median	1.48758	1.52467	1.60905	1.54501	
Rank sum	667	679	797	783	
H-stat					1.495737
H-ties					1.495757
df					3
p-value					0.68325

	Residuals
Mean	1.05179E-16
Standard Error	0.056227797
Median	-0.144415
Standard Deviation	0.490182568
Sample Variance	0.24027895
Kurtosis	5.801902378
Skewness	2.280540499
Range	2.57093
Maximum	1.977605789
Minimum	-0.593324211

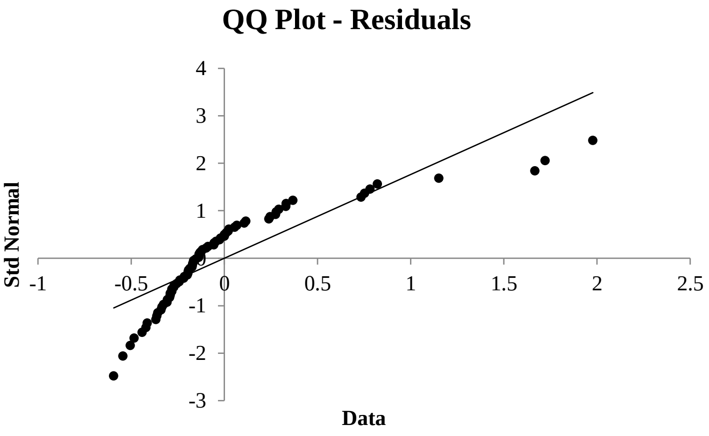

h. Relative head displacement (DWI): ANOVA, F=2.019, df=3,72, p=0.119. Shapiro-Wilk test for normality, W=0.988, p=0.696.

Groups	*Average*	*Variance*
C	0.505302	0.011806
CBS	0.442901	0.013825
PD	0.484848	0.021384
PSP-RS	0.421122	0.008483

ANOVA

	SS	*df*	*MS*	*F*	*P-value*	*F crit*
Between Groups	0.084044	3	0.028015	2.019188	0.118826	2.731807
Within Groups	0.998949	72	0.013874			
Total	1.082994	75				

	Residuals
Mean	6.93889E-17
Standard Error	0.013238363
Median	0.002924
Standard Deviation	0.115409374
Sample Variance	0.013319324
Kurtosis	0.337382356
Skewness	-0.308137695
Range	0.595293105
Maximum	0.244881632
Minimum	-0.350411474

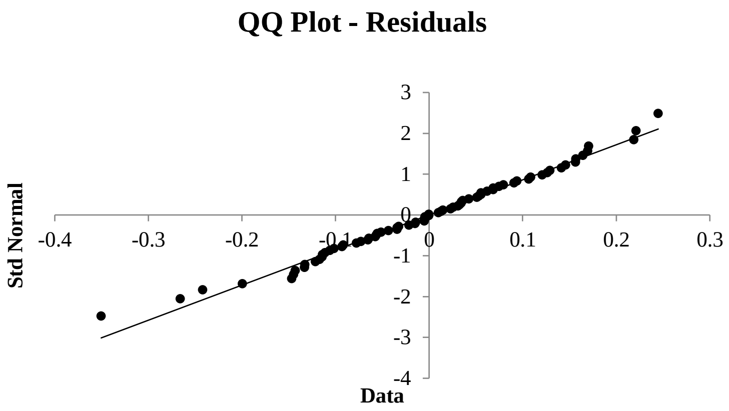

Independent validation group

i. Sex ratio: Chi-squared test, X^2=1.621, df=3, p=0.655.

j. Age: ANOVA, F=2.562, df=3,54, p=0.064. Shapiro-Wilk test for normality, W=0.985, p=0.686.

Groups	*Average*	*Variance*
C	69.70414	47.21061
PD	69.07177	44.88813
PSP-RS	70.90157	52.47892
CBS	62.22587	64.76825

ANOVA

	SS	*df*	*MS*	*F*	*P-value*	*F crit*
Between Groups	383.6601	3	127.8867	2.562159	0.064295	2.77576237
Within Groups	2695.337	54	49.91365			
Total	3078.997	57				

	Residuals
Mean	-4.65528E-15
Standard Error	0.902932146
Median	0.95515144
Standard Deviation	6.876526358
Sample Variance	47.28661475
Kurtosis	-0.114633889
Skewness	-0.362943242
Range	31.32586109
Maximum	13.21624025
Minimum	-18.10962084

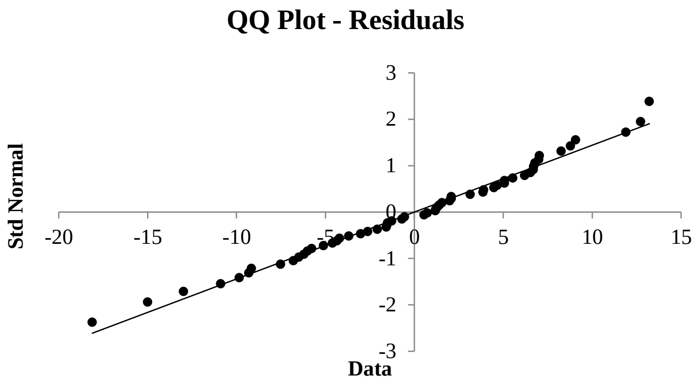

k. TIV: ANOVA, F=0.431, df=3,54, p=0.732. Shapiro-Wilk test for normality, W=0.967, p=0.111.

Groups	*Average*	*Variance*
C	739294.331	5913090897
PD	747752.825	4739776556
CBS	773393.363	5788663771
PSP-RS	731264.098	11450957696

ANOVA

	SS	*df*	*MS*	*F*	*P-value*	*F crit*
Between Groups	9009940778	3	3003313593	0.430785215	0.731784	2.775762
Within Groups	3.7647E+11	54	6971719296			
Total	3.8548E+11	57				

	Residuals
Mean	0.0000
Standard Error	10671.25263
Median	7783.0066
Standard Deviation	81269.83882
Sample Variance	6604786701
Kurtosis	-0.610523097
Skewness	0.102584538
Range	320123.4738
Maximum	179101.5701
Minimum	-141021.9038

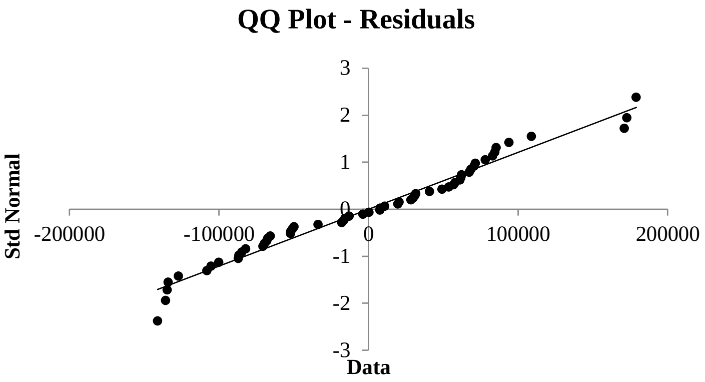

l. MPRAGE FWHM: Welch's ANOVA, F=2.362, df=3,19.3, p=0.103. Shapiro-Wilk test for normality, W=0.959, p=0.199.

Groups	*Average*	*Variance*
CBS	2061.195	43555.14
PSP-RS	2092.108	11398.47
PD	2048.944	27692.59
C	1989.588	14642.96

Welch's ANOVA Test	
F	2.362108
df1	3
df2	19.31827
P-value	0.102921

	Residuals
Mean	-7.4E-14
Standard Error	18.06262
Median	-27.133
Standard Deviation	137.5608
Sample Variance	18922.98
Kurtosis	-0.27288
Skewness	0.555956
Range	555.3508
Maximum	331.2806
Minimum	-224.07

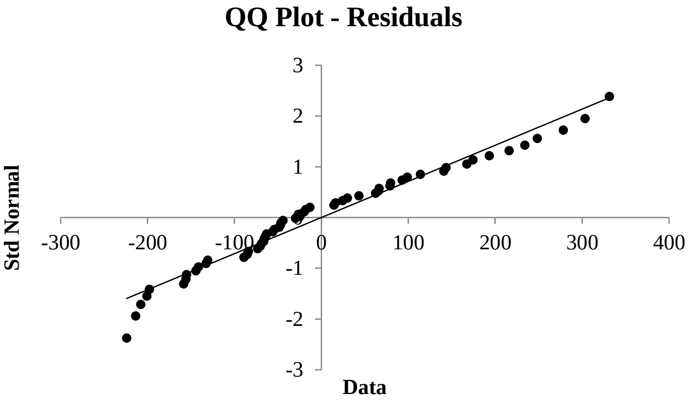

m. Absolute head displacement (DWI): Kruskal-Wallis ANOVA, H=5.849, df=3, p=0.119. Shapiro-Wilk test for normality, W=0.859, p<0.001.

Groups	*Average*	*Variance*
C	1.462518	0.033767
CBS	1.616876	0.083854
PD	1.466006	0.091695
PSP-RS	1.587008	0.047859

Kruskal-Wallis Test

	C	*CBS*	*PD*	*PSP-RS*	
Median	1.43678	1.51048	1.39593	1.56124	
Rank sum	648	252	300	511	
H-stat					5.849481
H-ties					5.849481
df					3
p-value					0.119167

	Residuals
Mean	-2.3E-16
Standard Error	0.030135
Median	-0.05281
Standard Deviation	0.229503
Sample Variance	0.052672
Kurtosis	4.864443
Skewness	1.797784
Range	1.33681
Maximum	0.974544
Minimum	-0.36227

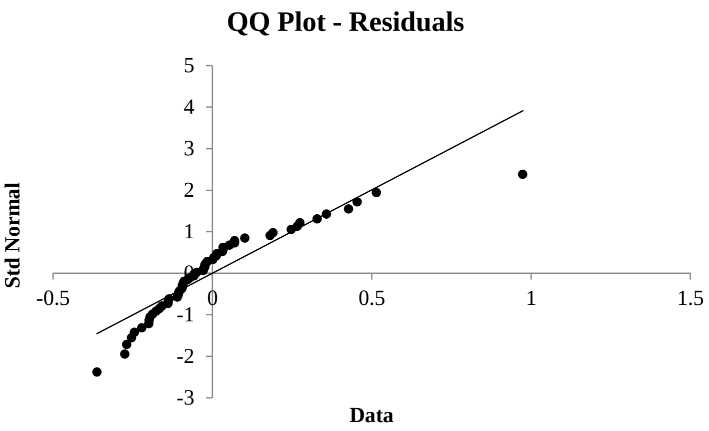

n. Relative head displacement (DWI): ANOVA, F=0.359, df=3,54, p=0.783. Shapiro-Wilk test for normality, W=0.988, p=0.819.

Groups	*Average*	*Variance*
C	0.476897	0.011974
CBS	0.482525	0.018679
PD	0.475045	0.006501
PSP-RS	0.444485	0.009139

ANOVA

Source of Variation	*SS*	*df*	*MS*	*F*	*P-value*	*F crit*
Between Groups	0.011659	3	0.003886	0.359177	0.7827	2.775762
Within Groups	0.584295	54	0.01082			
Total	0.595954	57				

	Residuals
Mean	-3.8E-18
Standard Error	0.013294
Median	0.001632
Standard Deviation	0.101246
Sample Variance	0.010251
Kurtosis	0.116568
Skewness	-0.184
Range	0.463071
Maximum	0.220528
Minimum	-0.24254

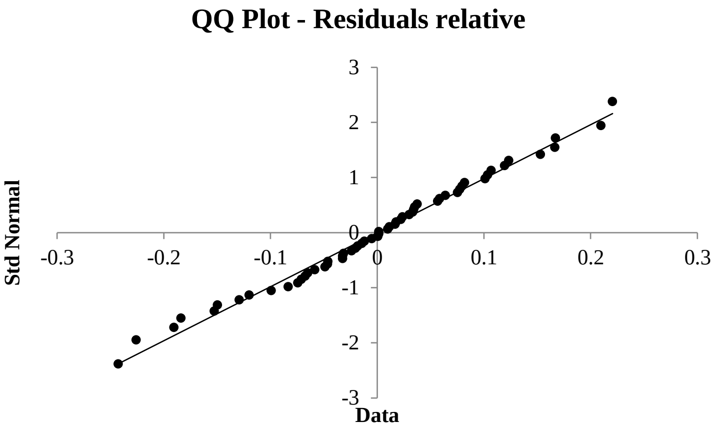

Section C – Summary motion metrics for datasets excluded after quality control

	**Controls**	**PD**	**PSP-RS**	**CBS**
Excluded by MPRAGE FWHM metric				
sample size	0	2	3	4
MPRAGE smoothness FWHM	--	2480.18±59.95 (2420.23-2540.13)	2628.85±211.5 (2373.19-3048.43)	2483.15±28.67 (2445.53-2539.43)
Excluded by DWI motion metrics				
sample size	7	3	19	10
Absolute head displacement (DWI)	2.38±0.13 (2.00-2.88)	2.54±0.24 (1.61-3.82)	2.56±0.24 (1.39-4.54)	2.75±0.27 (1.61-4.50)
Relative head displacement (DWI)	0.49±0.02 (0.40-0.55)	0.50±0.05 (0.32-0.71)	0.63±0.07 (0.29-1.43)	0.75±0.09 (0.39-1.61)

Section D – Comparison between GM atlases for extraction of cortical thickness (CT) features

In the main manuscript we presented cortical thickness results obtained with the cortical Harvard-Oxford atlas ROIs, after mapping of cortical thickness maps into MNI space. An alternative would be to use one of the atlases distributed with freesurfer (Desikan-Killiany atlas or Destrieux atlas), and extract the CT features in individual subject space. In this section we present a comparison of the results obtained with different atlases for feature extraction: Harvard-Oxford cortical atlas (as presented in the main manuscript), Desikan-Killiany atlas and Destrieux atlas.

The plots below show the accuracy results obtained with each of the three atlases. Box plots have been colour coded to reflect the atlas used for feature extraction, and each individual box represents one pairwise comparison between groups in the following order: C vs PD, C vs CBS, C vs PSP-RS, PD vs CBS, PD vs PSP-RS and CBS vs PSP-RS.

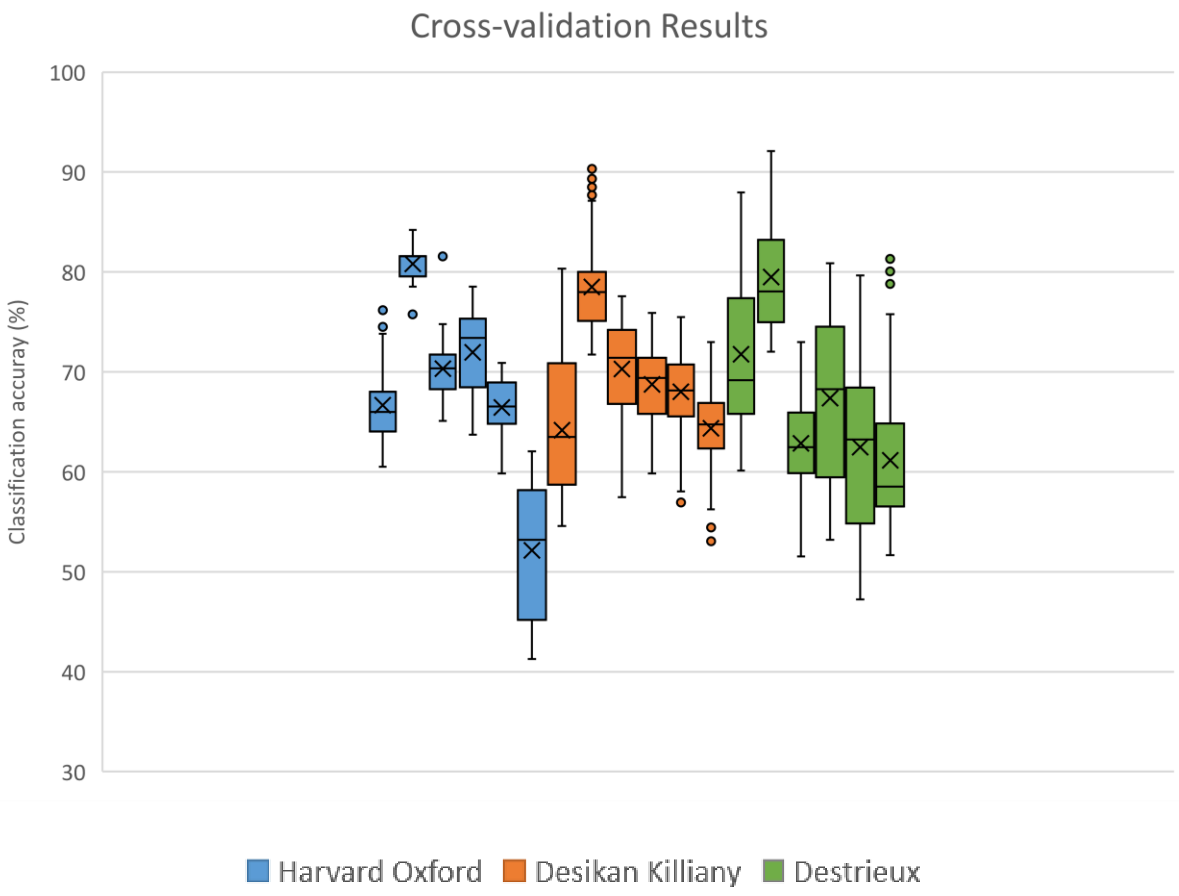

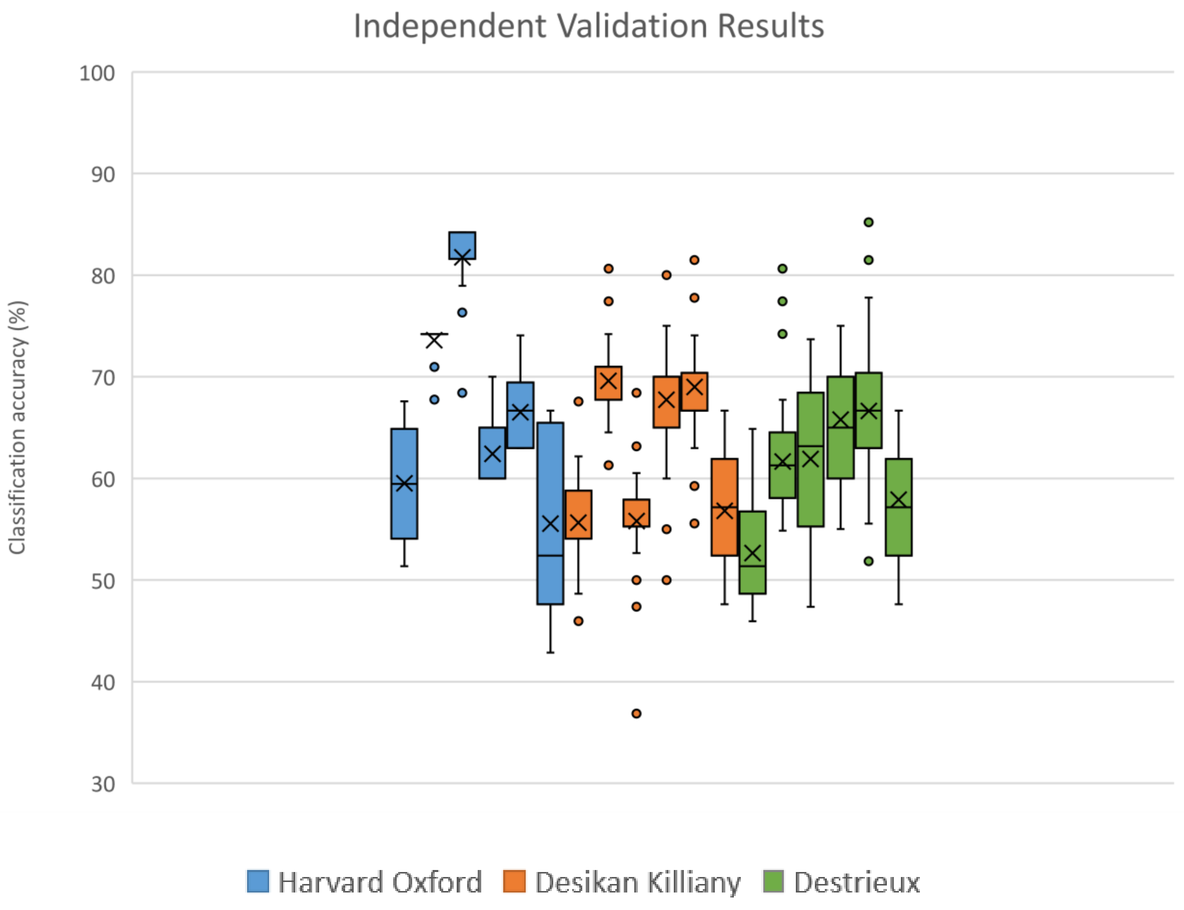

The results obtained with each of the three atlases are overall very comparable, but there are some notable differences. For the cross-validation analysis the accuracy obtained with the Harvard-Oxford atlas for the CBS vs PSP-RS pairwise comparison was noticeably lower than the accuracies obtained with the other two atlases, especially when compared to the Desikan-Killiany atlas. In contrast, for the validation analysis the Harvard-Oxford atlas clearly outperformed the other two for the pairwise comparisons C vs CBS and C vs PSP-RS.

Since none of the three atlases clearly outperformed the others for all pairwise comparisons and both validation approaches, we elected to use the Harvard-Oxford atlas in the main manuscript. This was to ensure a more direct comparability with the GM volume data.

Section E – Classification accuracy, sensitivity and specificity when cross-validation is used

The plots below show classification accuracy, sensitivity and specificity as a function of the number of features included in the model (number of ROIs or PCA components). Separate plots are also presented for T1-weighted and diffusion MRI data. Comparisons between controls and PSP-RS and between PD and PSP-RS are shown as representative examples.

T1-weighted data (GM volume maps) + ROIs

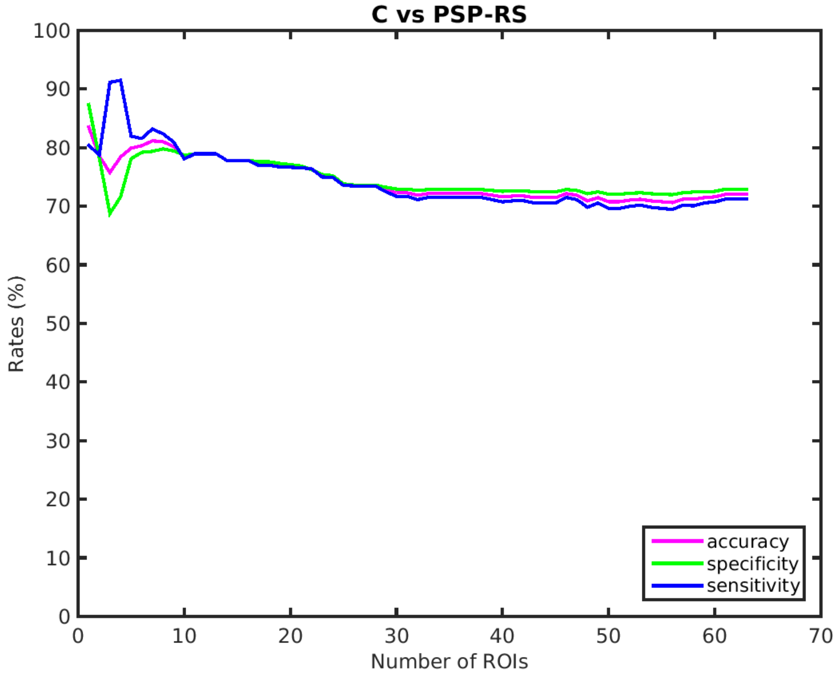

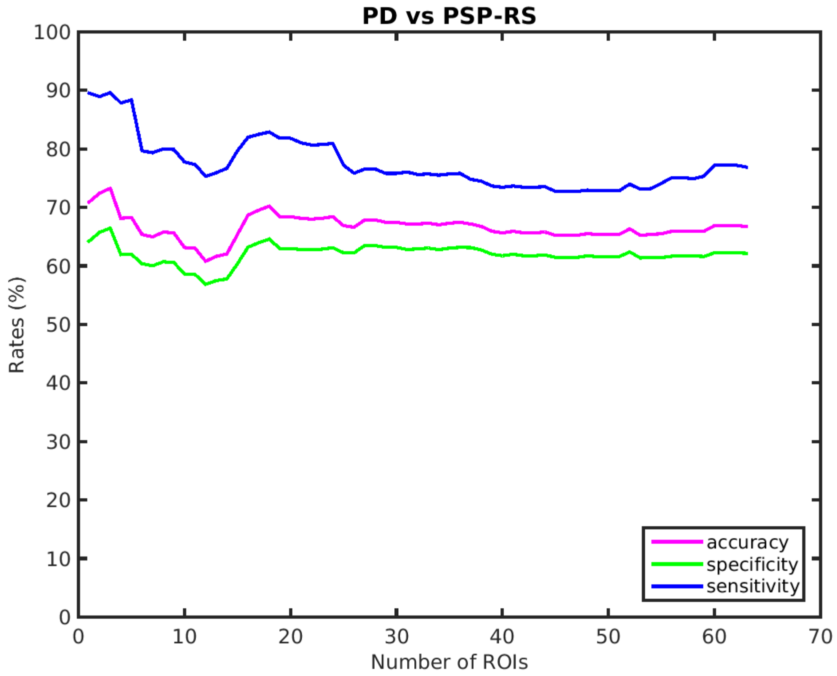

T1-weighted data (GM volume maps) + PCA

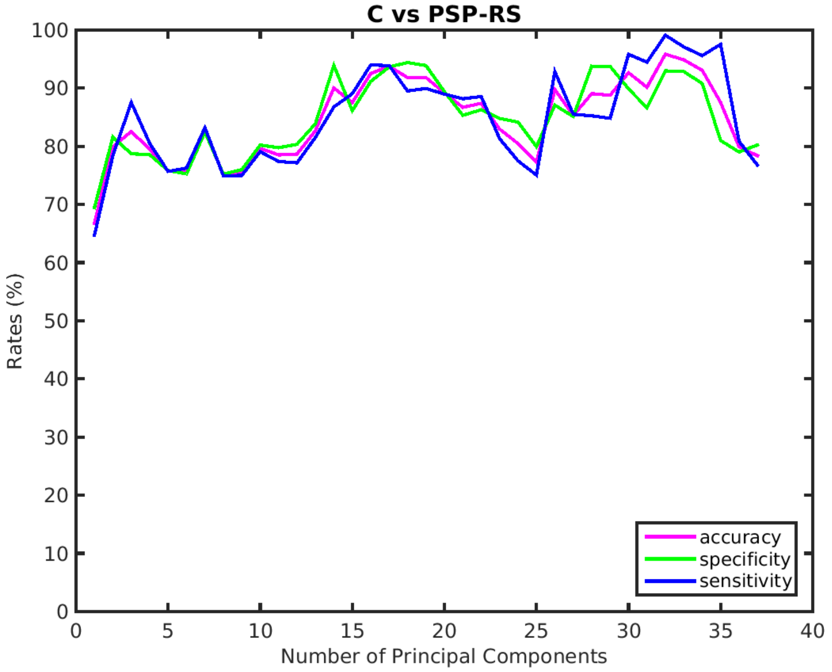

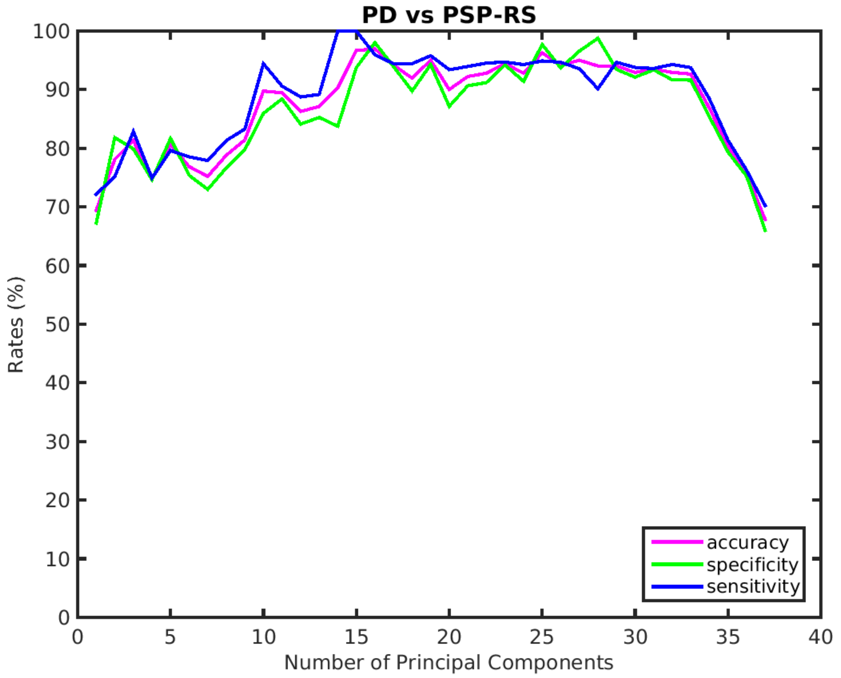

Diffusion MRI data (FA and MD maps) + ROIs

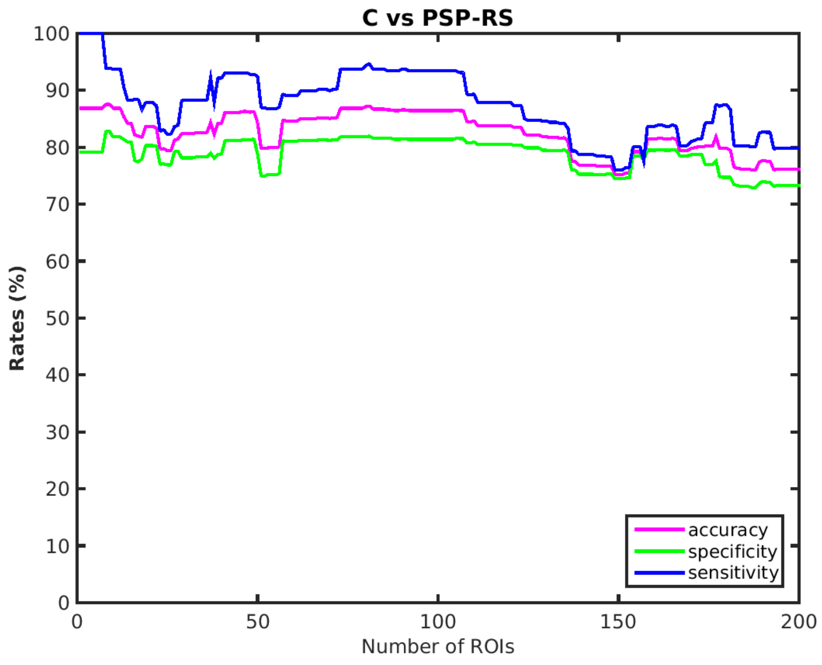

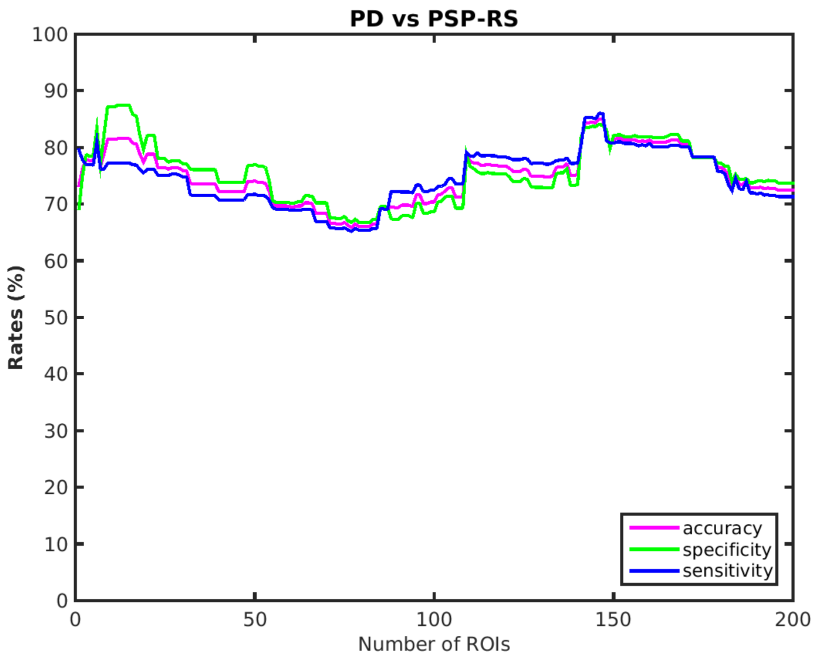

Diffusion MRI data (FA and MD maps) + PCA

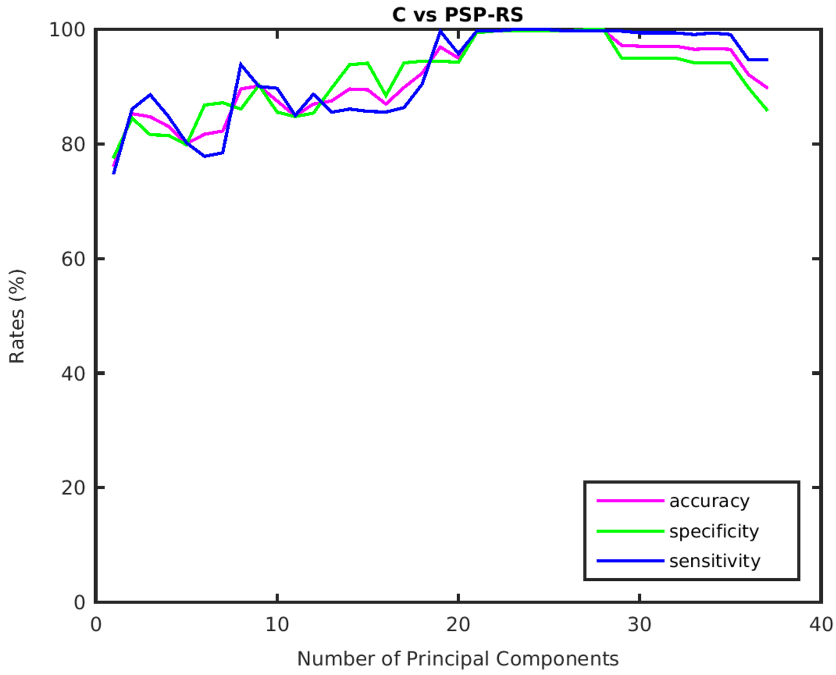

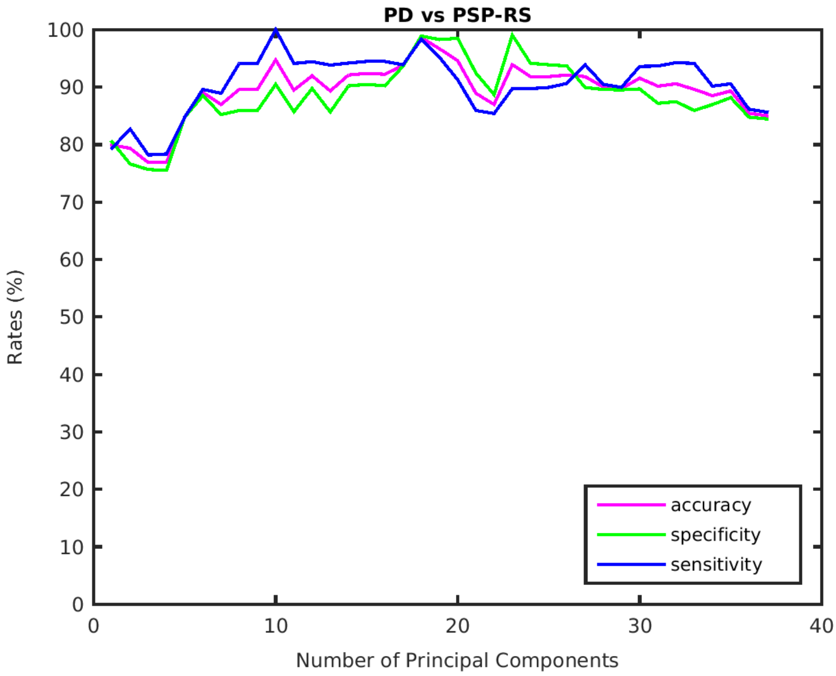

Section F – Classification accuracy, sensitivity and specificity when the independent validation group is used

The plots below show classification accuracy, sensitivity and specificity as a function of the number of features included in the model (number of ROIs or PCA components). Separate plots are also presented for T1-weighted and diffusion MRI data. Comparisons between controls and PSP-RS and between PD and PSP-RS are shown as representative examples.

<u>T1-weighted data (GM volume maps) + ROIs</u>

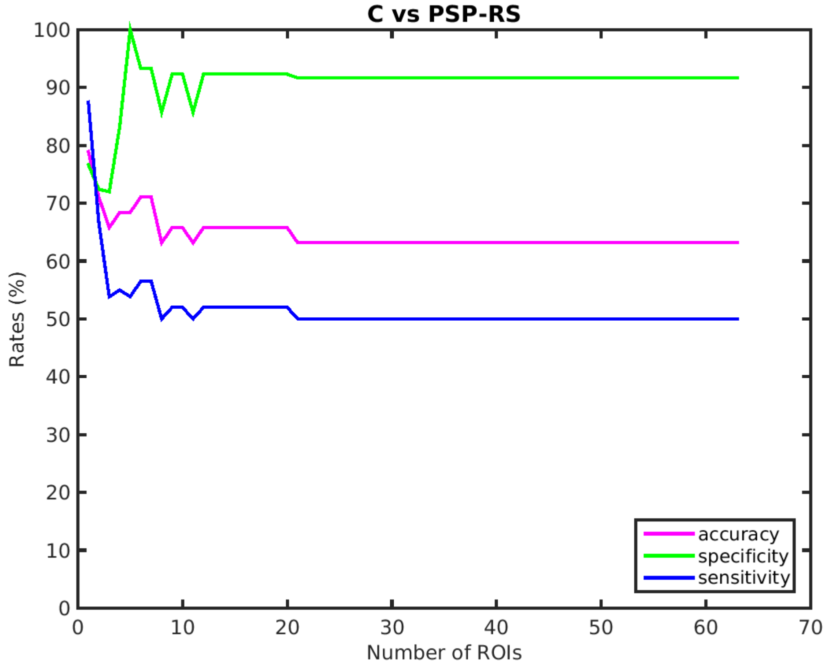

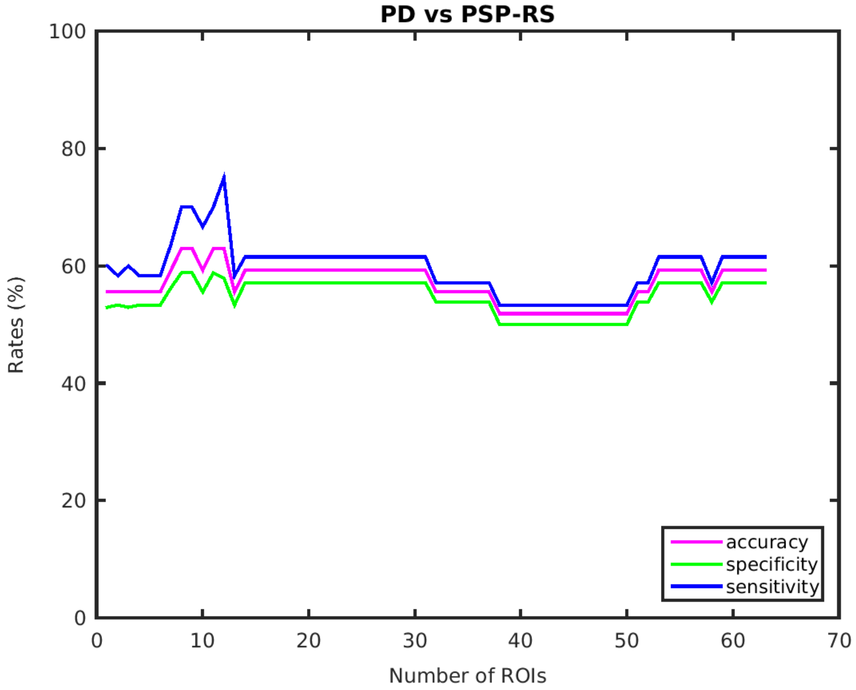

T1-weighted data (GM volume maps) + PCA

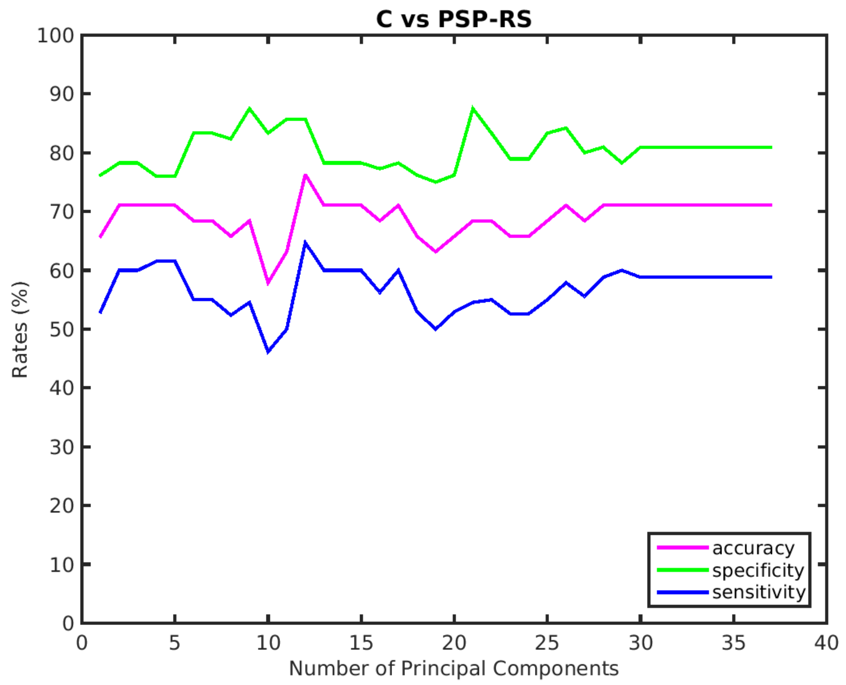

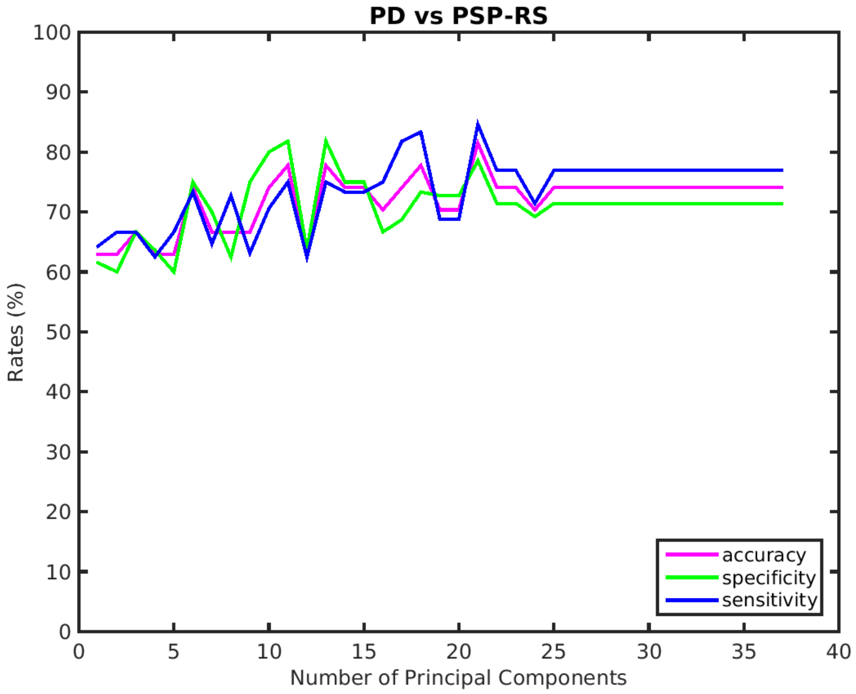

Diffusion MRI data (FA and MD maps) + ROIs

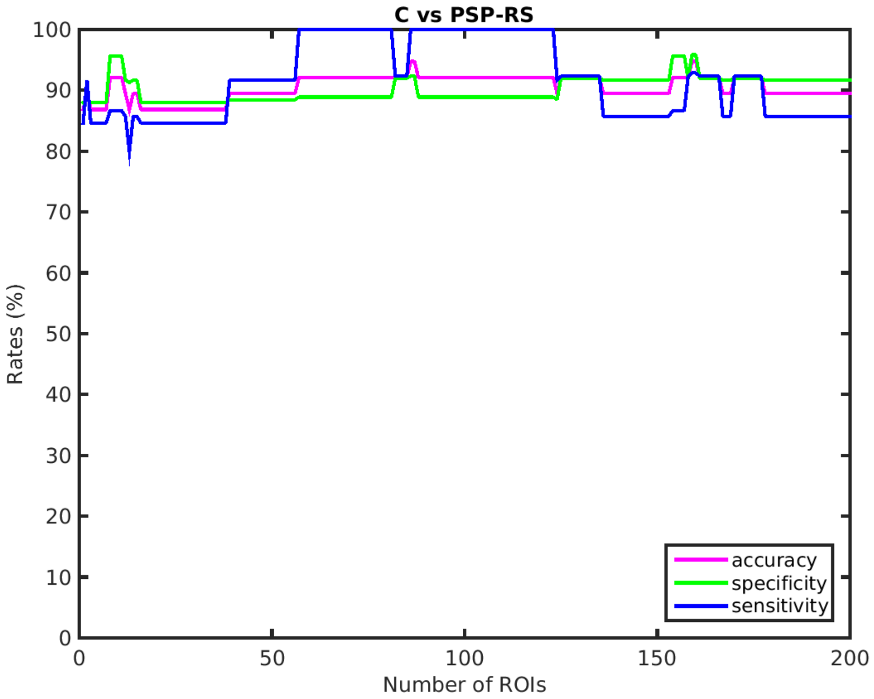

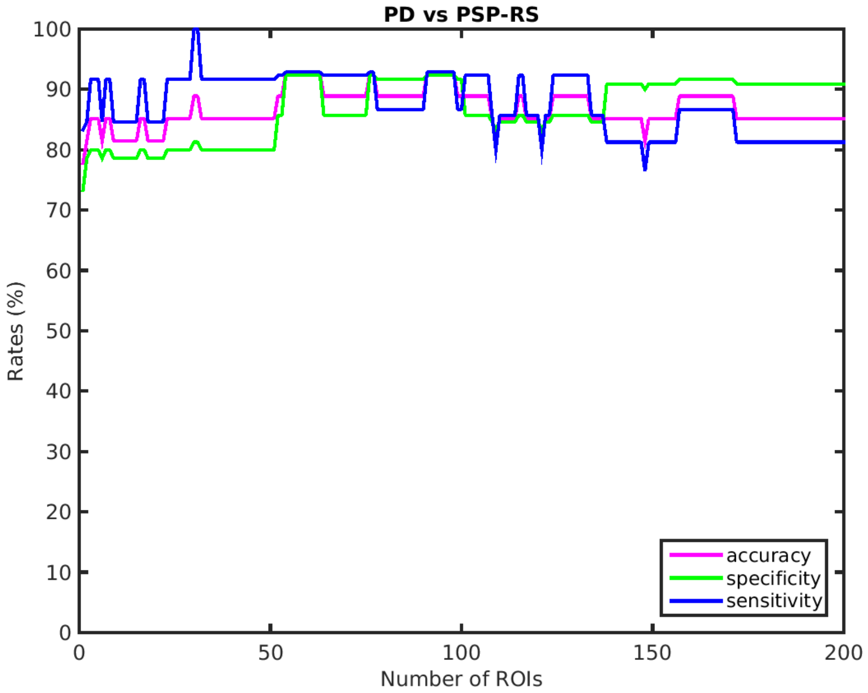

Diffusion MRI data (FA and MD maps) + PCA

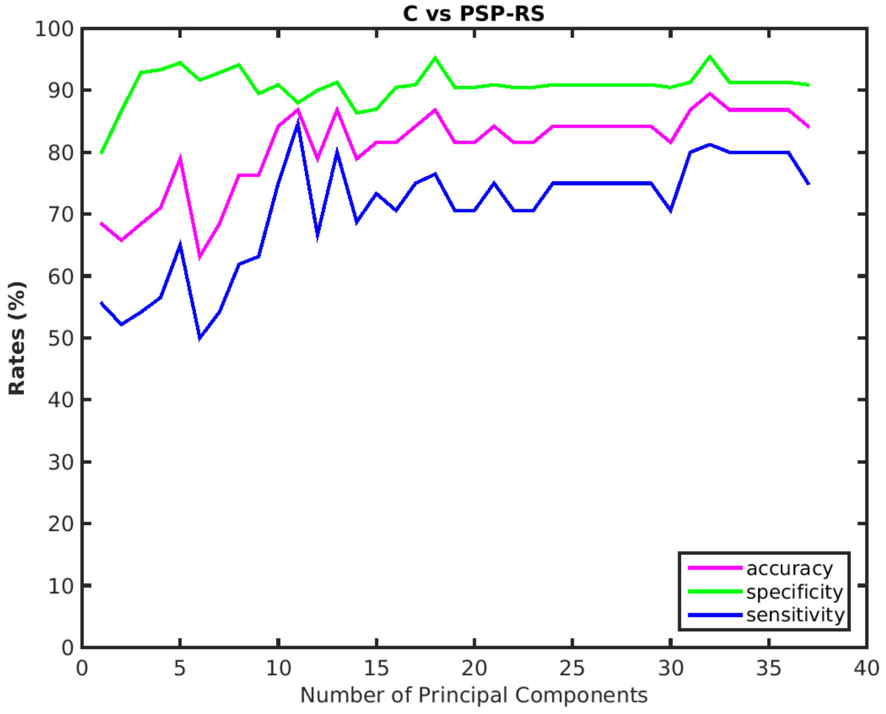

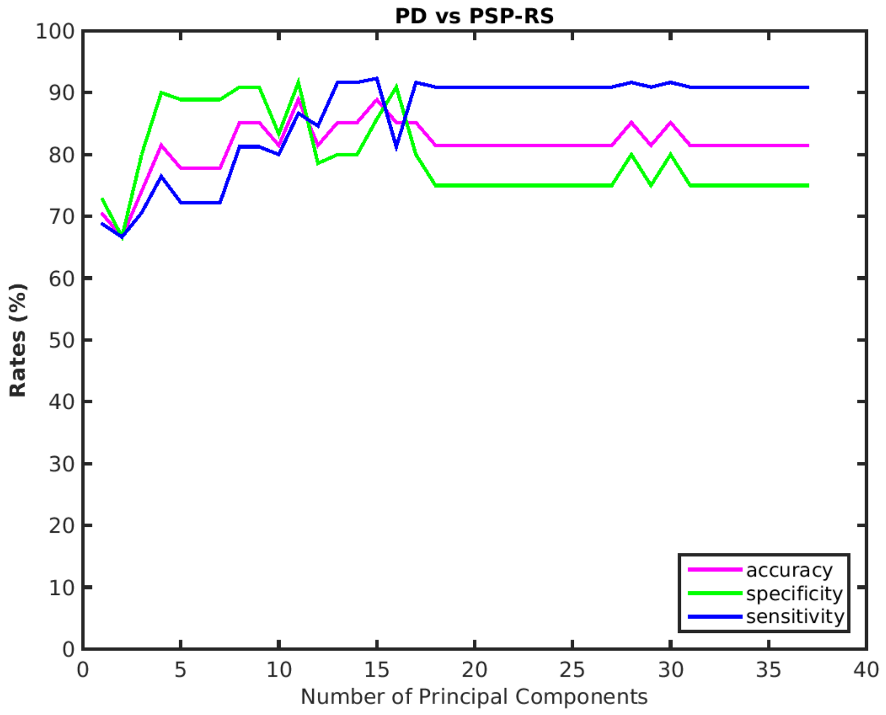

Section G – Comparison of cortical thickness (CT) and DTI metrics (FA+MD) for the independent validation cohort

The box plots below aim to provide a direct comparison between cortical thickness and DTI metrics for the independent validation sample. As discussed in the main manuscript, using CT as feature type was found to have better generalisation to independent data when compared to GM volume. However, when compared directly to the results obtained with the DTI metrics, the plots below show that DTI metrics clearly outperform CT for three out of the six pairwise comparisons (C vs CBS, C vs PSP-RS and PD vs CBS), resulting in higher classification accuracies when independent data is used to assess the generalisability of the statistical model.

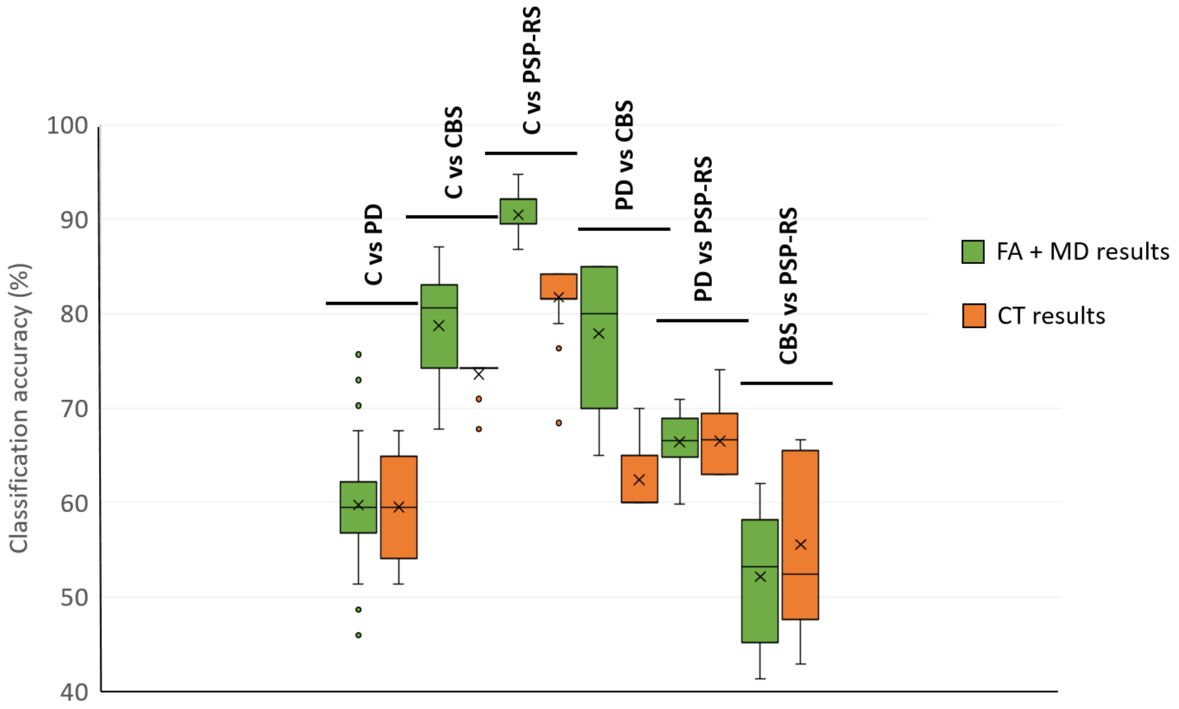

Section H – Comparison of classification accuracy for different DTI metrics

The tables below show a comparison between the classification results obtained when FA and/or MD are used as features for classification. For both mean and maximum accuracy, the highest value across the three modalities (FA+MD, FA only and MD only) has been highlighted in green. However, it should be noted that in many cases, the difference between modalities is very small. Sample plots for classification accuracy as a function of the number of features are also shown for two of the group pairwise comparisons as representative examples.

ROIs – cross-validation

	Mean classification accuracy (IQR) (%)			Maximum classification accuracy (%)		
	**FA+MD**	**FA only**	**MD only**	**FA+MD**	**FA only**	**MD only**
**C vs PD**	61.26 (12.88)	54.66 (17.00)	66.64 (4.74)	75.21	80.47	76.87
**C vs CBS**	70.13 (5.54)	72.46 (2.77)	67.75 (4.71)	77.29	81.44	75.07
**C vs PSP-RS**	82.45 (6.44)	80.59 (1.32)	78.46 (10.25)	87.53	91.27	88.50
**PD vs CBS**	72.89 (10.66)	68.23 (0.83)	68.18 (4.43)	81.99	72.85	77.01
**PD vs PSP-RS**	74.93 (6.58)	79.56 (2.11)	71.27 (6.41)	85.04	85.73	79.09
**CBS vs PSP-RS**	79.84 (7.89)	72.98 (7.13)	79.00 (5.44)	90.72	81.03	90.17

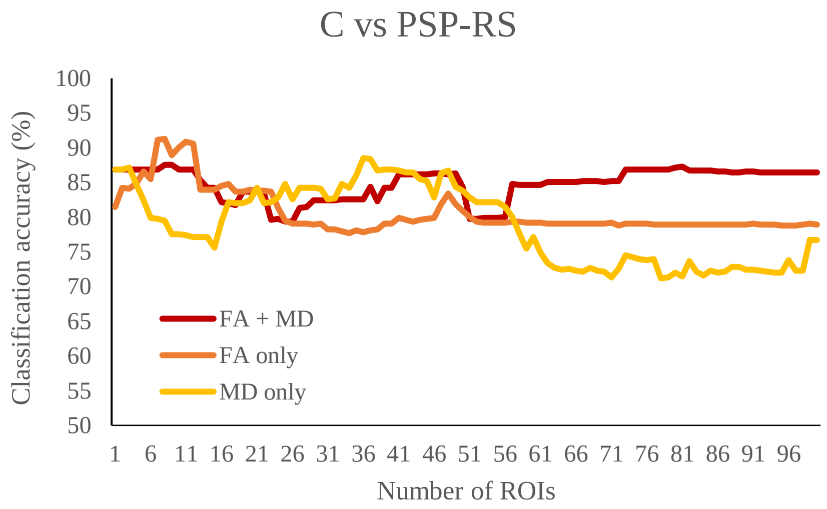

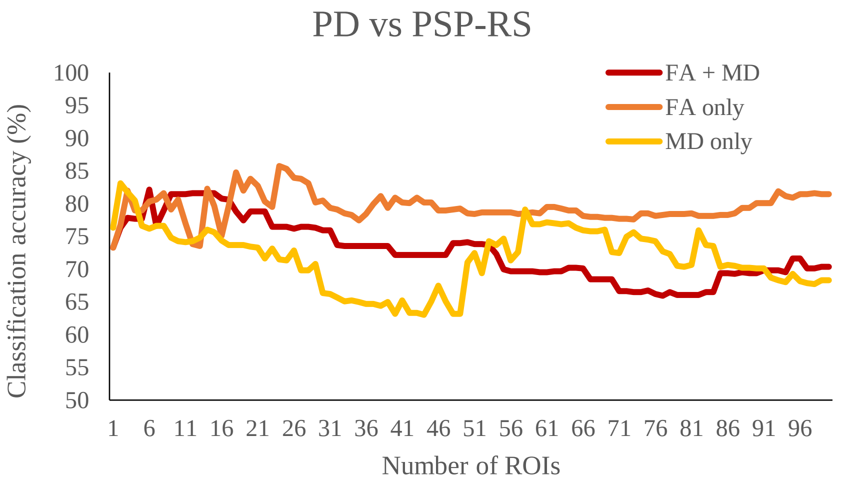

ROIs – independent validation

	Mean classification accuracy (IQR) (%)			Maximum classification accuracy (%)		
	**FA+MD**	**FA only**	**MD only**	**FA+MD**	**FA only**	**MD only**
**C vs PD**	59.74 (5.41)	44.05 (5.41)	59.51 (5.41)	75.68	67.57	75.68
**C vs CBS**	78.74 (5.37)	85.13 (3.23)	77.87 (6.45)	87.09	90.32	87.09
**C vs PSP-RS**	90.49 (2.63)	85.34 (2.63)	90.53 (2.63)	94.74	89.47	94.74
**PD vs CBS**	77.90 (15.0)	70.65 (5.00)	77.20 (15.0)	85.00	75.00	85.00
**PD vs PSP-RS**	86.78 (3.70)	76.37 (3.70)	86.63 (3.70)	92.59	85.19	92.59
**CBS vs PSP-RS**	76.33 (4.76)	72.19 (0.00)	76.71 (4.76)	80.95	76.19	80.95

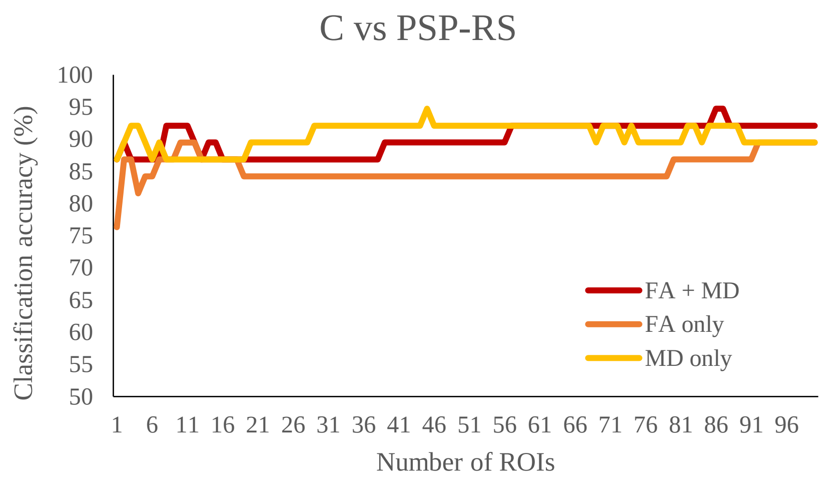

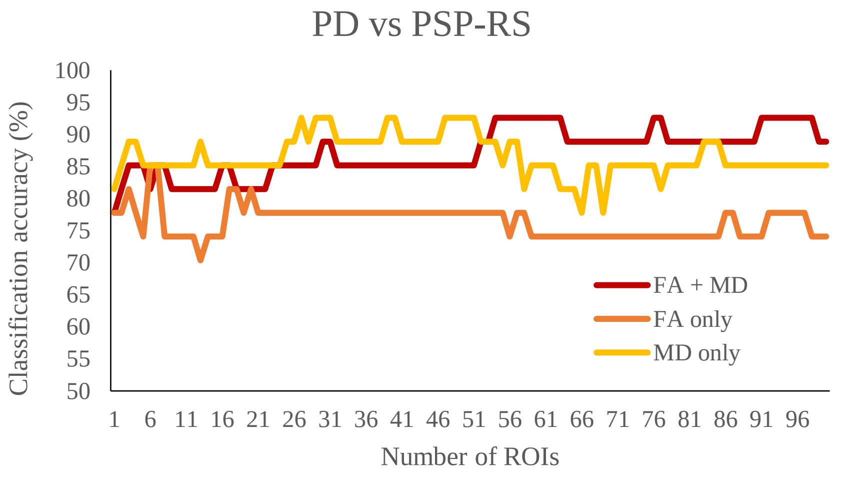

PCA – cross-validation

	Mean classification accuracy (IQR) (%)			Maximum classification accuracy (%)		
	FA+MD	FA only	MD only	FA+MD	FA only	MD only
**C vs PD**	85.43 (19.43)	84.45 (14.26)	86.69 (12.60)	99.72	99.72	99.58
**C vs CBS**	90.06 (9.90)	84.38 (11.63)	85.27 (14.96)	97.37	96.68	97.23
**C vs PSP-RS**	92.51 (10.15)	94.45 (10.39)	90.72 (9.70)	99.86	100.0	99.44
**PD vs CBS**	84.40 (7.34)	85.34 (10.94)	81.46 (6.51)	91.55	98.48	91.27
**PD vs PSP-RS**	89.49 (3.98)	89.80 (11.08)	90.08 (8.31)	96.40	99.72	99.45
**CBS vs PSP-RS**	94.95 (12.67)	93.66 (9.97)	89.50 (12.47)	100.0	100.0	99.72

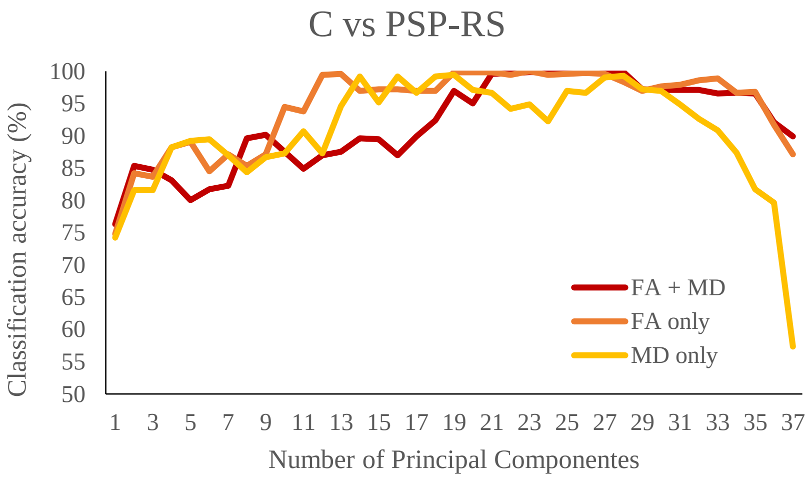

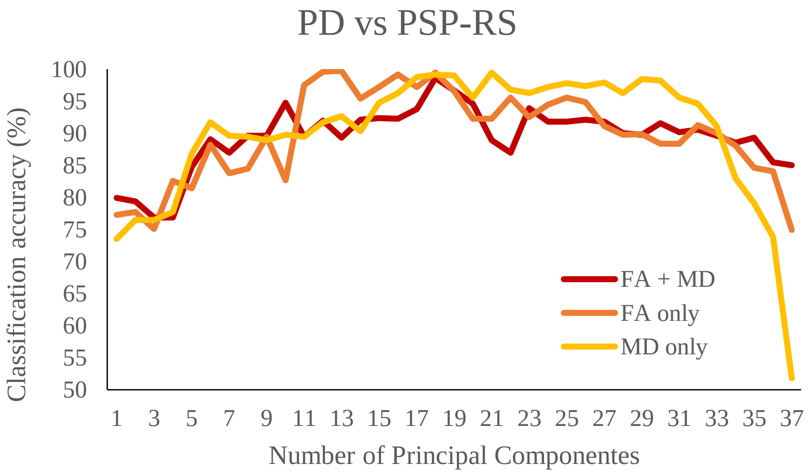

PCA – independent validation

	Mean classification accuracy (IQR) (%)			Maximum classification accuracy (%)		
	FA+MD	FA only	MD only	FA+MD	FA only	MD only
**C vs PD**	57.63 (10.81)	64.65 (8.11)	61.87 (2.70)	72.97	78.39	72.97
**C vs CBS**	73.41 (6.45)	70.53 (6.45)	73.67 (6.45)	80.64	90.32	80.65
**C vs PSP-RS**	80.87 (5.92)	81.08 (7.90)	80.51 (5.26)	89.44	86.84	89.44
**PD vs CBS**	80.81 (5.00)	80.14 (5.00)	80.27 (10.00)	85.00	90.00	90.00
**PD vs PSP-RS**	81.48 (3.70)	84.18 (0.00)	81.48 (3.70)	88.89	88.89	92.59
**CBS vs PSP-RS**	80.82 (4.95)	68.98 (4.76)	76.57 (9.52)	90.63	76.19	90.48

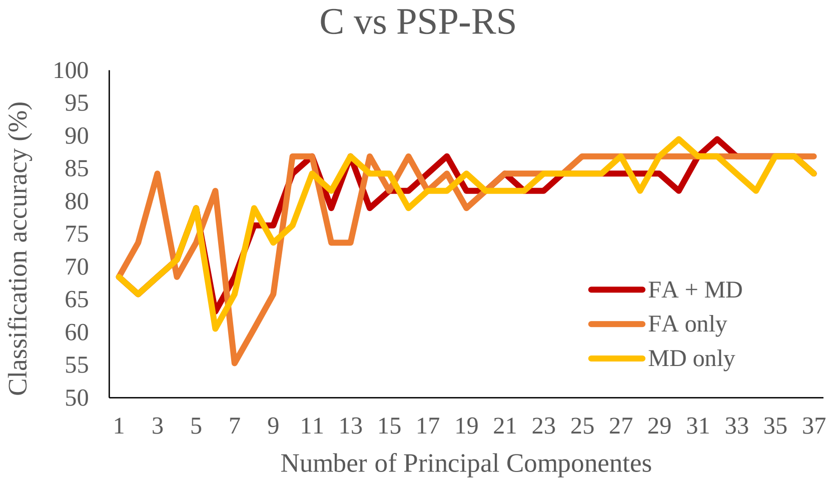

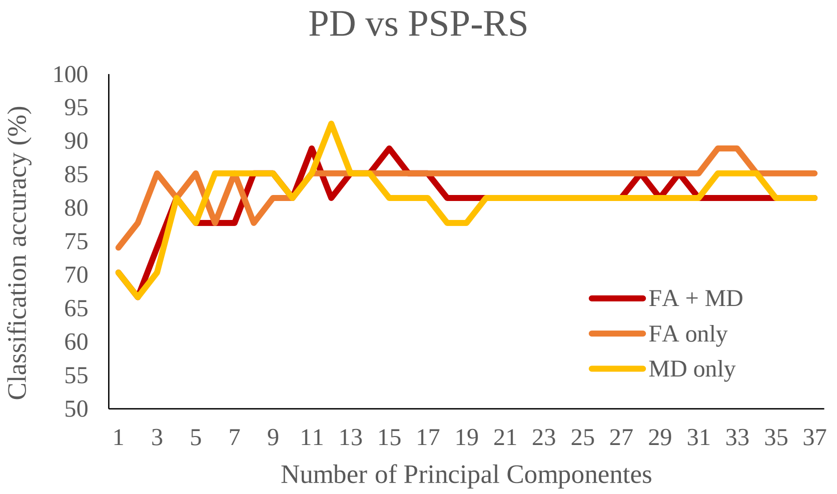

Section I – Classification accuracies including all GM, FA and MD ROI features

ROIs – cross-validation

	Mean classification accuracy (IQR) (%)			Maximum classification accuracy (%)		
	FA+MD	GM	GM+FA+MD	FA+MD	GM	GM+FA+MD
**C vs PD**	61.26 (12.88)	71.96 (2.18)	68.44 (6.10)	75.21	85.46	82.83
**C vs CBS**	70.13 (5.54)	83.36 (0.59)	80.95 (1.94)	77.29	91.69	90.58
**C vs PSP-RS**	82.45 (6.44)	73.74 (5.68)	86.01 (2.22)	87.53	81.02	92.11
**PD vs CBS**	72.89 (10.66)	77.93 (3.88)	74.23 (4.71)	81.99	85.87	90.03
**PD vs PSP-RS**	74.93 (6.58)	67.44 (2.35)	79.79 (2.91)	85.04	70.91	85.60
**CBS vs PSP-RS**	79.84 (7.89)	62.16 (0.55)	67.09 (2.08)	90.72	65.65	76.04

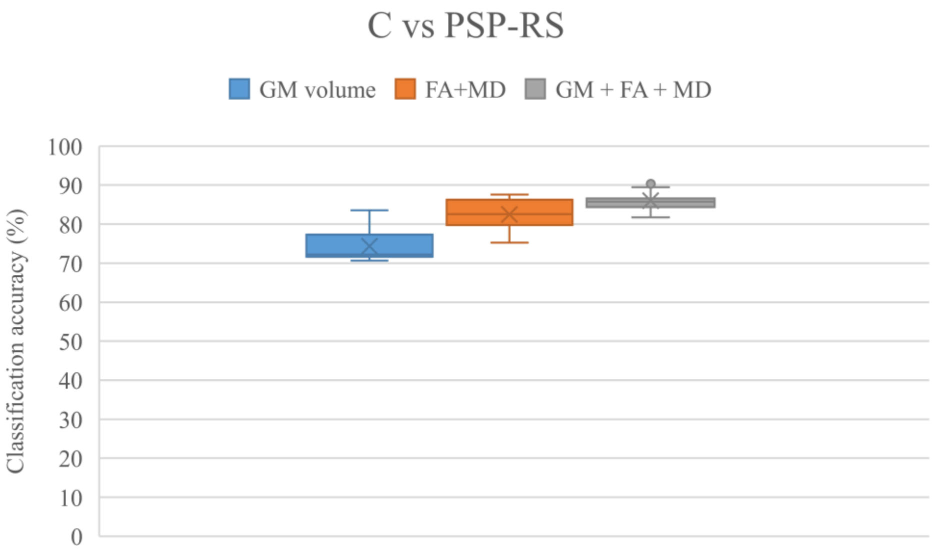

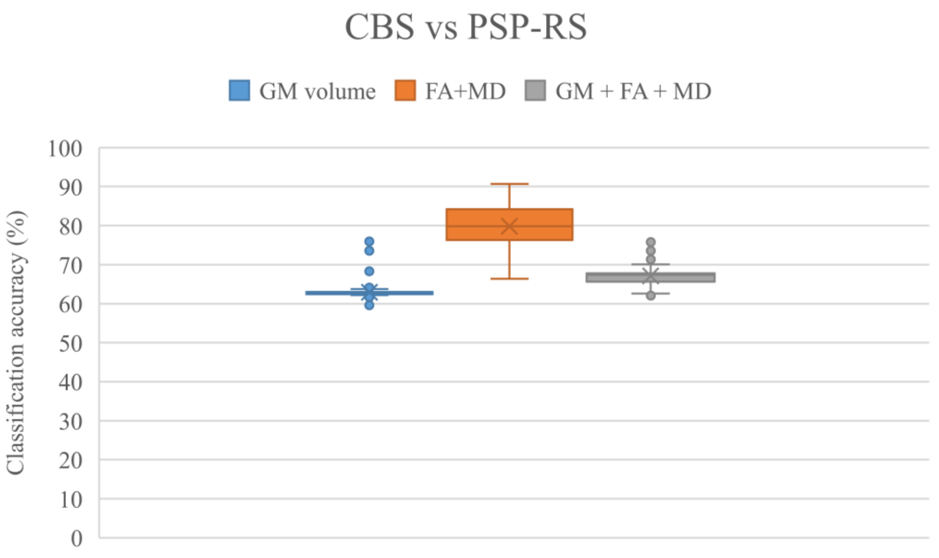

ROIs – independent validation

	Mean classification accuracy (IQR) (%)			Maximum classification accuracy (%)		
	**FA+MD**	**GM**	**GM+FA+MD**	**FA+MD**	**GM**	**GM+FA+MD**
**C vs PD**	59.74 (5.41)	47.75 (8.11)	50.95 (2.70)	75.68	64.86	64.87
**C vs CBS**	78.74 (5.37)	63.95 (6.45)	66.93 (3.23)	87.09	74.19	74.19
**C vs PSP-RS**	90.49 (2.63)	62.78 (4.61)	81.50 (0.00)	94.74	76.32	92.11
**PD vs CBS**	77.90 (15.0)	47.14 (8.75)	59.63 (0.00)	85.00	60.00	65.00
**PD vs PSP-RS**	86.78 (3.70)	57.67 (3.70)	75.28 (3.70)	92.59	62.96	85.19
**CBS vs PSP-RS**	76.33 (4.76)	44.37 (4.76)	48.96 (0.00)	80.95	61.90	76.19

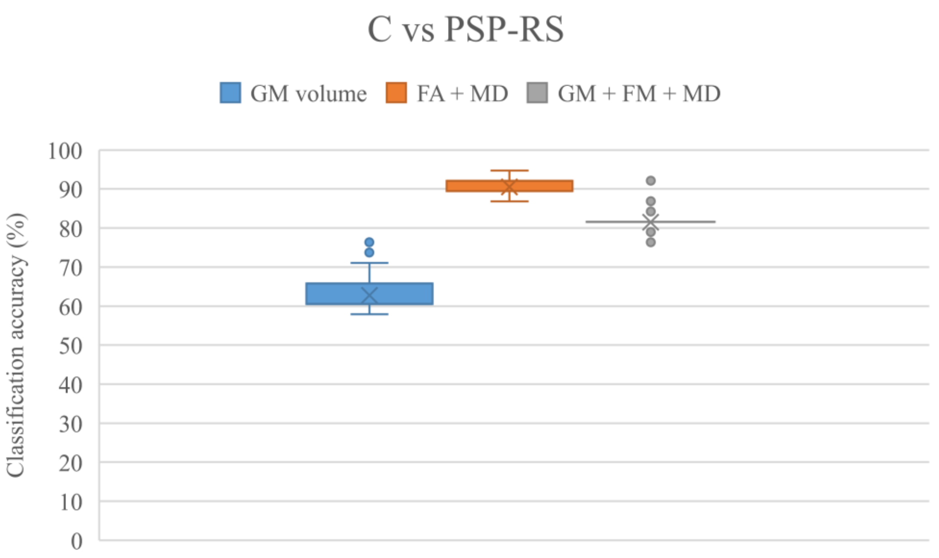

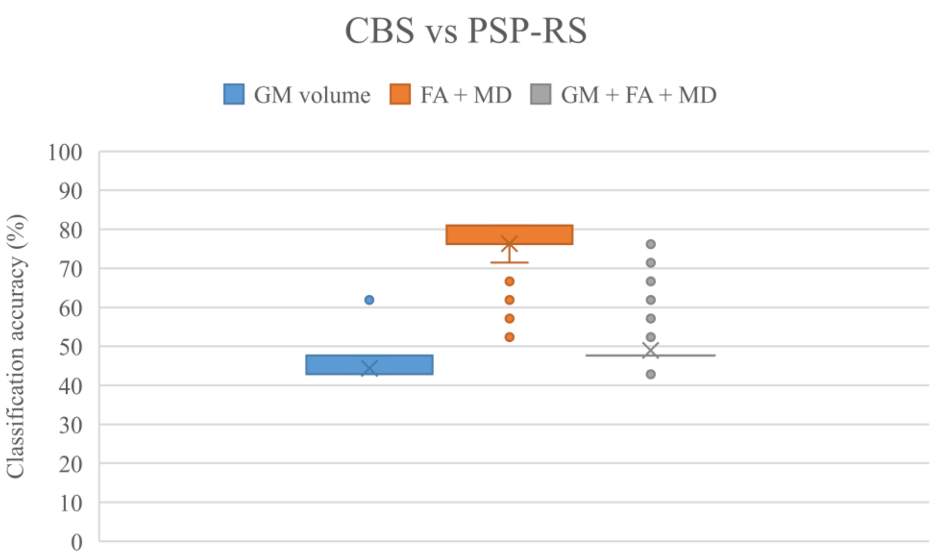

References

George, D., & Mallery, M. (2010). SPSS for Windows Step by Step: A Simple Guide and Reference, 17.0 update (10a ed.). Boston: Pearson.

Dean, A. M., and Voss, D. (1999). Design and Analysis of Experiments, page 112. Springer.
